# A novel LepR knockout hamster model for type 2 diabetes and its complications: a complementary platform to conventional rodent models

**DOI:** 10.3389/fendo.2026.1866850

**Published:** 2026-08-14

**Authors:** Xijin Tang, Jiahui Lu, Liuting Long, Minghong Huang, Wentao Zeng, Aihua Zhang, Qin Chen, Aimin Shi, Jianmin Li, Yun Gao

**Affiliations:** State Key Laboratory of Reproductive Medicine and Offspring Health, Jiangsu Laboratory Animal Center, Animal Core facility, Key Laboratory of Model Animal, Collaborative Innovation Center for Cardiovascular Disease Translational Medicine, National Vaccine Innovation Platform, Nanjing Medical University, Nanjing, China

**Keywords:** diabetic nephropathy, disease model, dyslipidemia, golden hamster, leptin receptor, metabolic dysfunction-associated steatotic liver disease, type 2 diabetes

## Abstract

**Background:**

Type 2 diabetes (T2D) is a progressive metabolic disorder characterized by systemic metabolic dysfunction and multi-organ complications that considerably increase morbidity and mortality. Leptin signaling via leptin receptor (LepR) is vital for maintaining energy homeostasis and insulin sensitivity; its disruption is strongly associated with obesity-induced T2D. However, currently available rodent models cannot effectively mimic the critical features of T2D in humans, particularly dyslipidemia, hypertension, and progressive diabetic nephropathy (DN). Lipoprotein metabolism in golden hamsters (*Mesocricetus auratus*) is similar to that in humans; therefore, they can serve as a potentially improved model for metabolic disease.

**Methods:**

We generated a *LepR* knockout golden hamster model using CRISPR/Cas9 genome editing to further investigate the role of LepR in T2D pathogenesis.

**Results:**

LepR*-*deficient (*db/db*) golden hamsters developed progressive obesity, sustained hyperglycemia, impaired glucose clearance, insulin resistance, and marked dyslipidemia, including elevated total cholesterol and low-density lipoprotein cholesterol levels. Notably, the model developed metabolic dysfunction-associated steatotic liver disease (MASLD) with early fibrotic remodeling, spontaneous hypertension, and early-stage renal injury characterized by proteinuria, impaired glomerular filtration barrier integrity, and interstitial fibrosis. Transcriptomic profiling of the adipose tissue, liver, and kidney revealed the coordinated activation of the inflammatory pathway and dysregulation of lipid metabolism and extracellular matrix remodeling. Cross-species integration analysis with human DN datasets revealed conserved transcriptional programs, thereby supporting the relevance of this model in human diseases.

**Conclusion:**

The LepR-deficient golden hamster model replicates key human T2D-associated metabolic, hepatic and renal phenotypes, providing a complementary preclinical platform for mechanistic studies of diabetes and its complications.

## 1 Introduction

Current epidemiological data suggest that more than half a billion people worldwide have diabetes (1). Approximately 96% of diagnosed diabetes cases are type 2 diabetes (T2D) (2).

As a multifactorial, heterogeneous metabolic disorder, T2D is caused primarily by interactions among genetic, environmental, and other risk factors, including obesity (3). Each of these risk factors ultimately results in insulin resistance, leading to hyperglycemia (4, 5). Chronic hyperglycemia induces systemic vascular damage that affects multiple organs, including the heart, liver, and kidneys, ultimately resulting in various complications and increasing the risk of mortality (6–9). Diabetic nephropathy (DN) is a life-threatening complication of diabetes, affecting one-third of patients and significantly contributing to cardiovascular morbidity and mortality (10, 11).

Many animal models have been constructed to investigate the prevention, diagnosis, and treatment of T2D. These can be broadly categorized into two types: spontaneous models, including *db/db* mice, *ob/ob* mice, and Zucker diabetic fatty rats, and induced models, which are generated via environmental or chemical interventions (12–15). However, existing animal models of diabetes differ substantially from those of T2D in terms of various aspects. Previous studies suggest that *db/db* mice exhibit severe, early-onset obesity, whereas patients with T2D show variable, less severe obesity that can occur at any age and rarely reaches the severity seen in early-life rodent models (12, 16).

Although hyperglycemia is a hallmark of T2D, glucose clearance is decreased in patients but increased in *db/db* mice (17, 18). Microvascular and macrovascular complications, including DN, are the primary drivers of morbidity and mortality in T2D patients. Unlike humans, *db/db* mice do not develop hypertension, a crucial cofactor in renal injury; furthermore, they exhibit only mild proteinuria, which is detectable only at later stages (17). Moreover, *db/db* mice incompletely model advanced nephropathic features, including significant glomerular basement membrane thickening or severe renal interstitial fibrosis (19–22). Recognizing these differences, we sought to establish a complementary model for further mechanistic investigations of T2D.

Golden hamsters (*Mesocricetus auratus*) offer significant advantages for metabolic research, particularly with respect to energy and lipid homeostasis. Their adipose tissue exhibits strong thermogenic potential (23, 24). In particular, brown adipose tissue expresses adrenergic receptors, facilitating UCP1-mediated non-shivering thermogenesis in response to cold (25–27). Moreover, like humans, golden hamsters possess cholesterol ester transfer protein (CETP) and peroxisome proliferator-activated receptors, as well as enzymatic pathways in lipoprotein and bile metabolism similar to those observed in humans (28–32). These features facilitate the precise modeling of human lipid metabolism, overcoming the crucial limitation of commonly used rodent models, such as mice and rats, that lack these features.

Despite the substantial translational value of golden hamsters in physiological and metabolic research, their broader application is limited by challenges in genetic manipulation. Through the systematic optimization of key procedures—including embryo handling, microinjection, and cryopreservation—we have overcome these technical barriers and achieved the efficient and stable production of transgenic golden hamsters (33).

In the present study, we used a leptin receptor (*LepR*) knockout (*db/db)* golden hamster model to investigate the role of LepR in T2D pathogenesis and its regulatory mechanisms. We systematically dissected the effects of LepR deficiency on lipid metabolism, liver function, and kidney injury across five key tissues (three adipose depots, liver, and kidney) and compared these findings with those from existing rodent models and clinical data. Given the increasing morbidity of T2D and its complications, this model serves as a complementary resource, offering a valuable dimension for assessing disease severity and investigating the underlying pathogenesis of diabetes.

## 2 Materials and methods

### 2.1 Animals

The *LepR*-knockout golden hamsters used in this study were generated by our research team using CRISPR/Cas9-mediated genome editing, as previously described (34), and were backcrossed for at least eight generations before experimental use. This study was approved by the Animal Ethics and Welfare Committee of Nanjing Medical University (No. IACUC-2310111) and was conducted in accordance with the Guide for the Care and Use of Laboratory Animals. The golden hamsters were maintained on a normal chow diet (NCD) throughout the experiment. All the cages were identical in size and material. The animals were housed in a climate-controlled facility (20–26 °C, 30–70% humidity, 14 h light:10 h dark cycle). As body weight trends were similar between the sexes in preliminary tests, subsequent experiments used only males.

### 2.2 Genotyping of *db/db* golden hamsters

Genomic DNA from *db/db* golden hamsters and WT controls was genotyped using a commercial one-step genotyping kit (Vazyme, PD101-01), followed by PCR amplification with flanking primers (forward: 5’-GTTTGGTGGATGAATCTAGCTGAGAA-3’; reverse: 5’-ACTCTAGCACAGTCTGTGACAAGT-3’).

### 2.3 Glucose tolerance test and insulin tolerance test

For GTT, WT and *db/db* hamsters (8-week-old, n=5; 18-week-old, n=3) were fasted for 16 hours (from 5 p.m. to 9 a.m.). 20% glucose (2 g/kg) was injected intraperitoneally, and blood glucose levels were measured at 0 min and 15, 30, 60, 90, and 120 min after glucose administration. For ITT, WT and *db/db* hamsters (8-week-old, n=5; 18-week-old, n=3) were fasted for 4 h (from 10 a.m. to 14 p.m.). Recombinant human insulin (0.75 U/kg) was administered intraperitoneally. Blood glucose levels were measured at 0 min and 15, 30, 60, 90, and 120 min after insulin administration. Blood glucose levels were measured from the retro orbital sinus using a glucose meter (Yuzhun 1, Yuyue).

### 2.4 Metabolic rate and physical activity

8-week-old WT and *db/db* hamsters (n=3) were acclimated to the TSE system (TSE Phenomaster) for 24 hours, and then oxygen consumption (VO_2_) and carbon dioxide production (VCO_2_) were measured over the subsequent 24 hours, normalized to body weight, and expressed as ml/h/kg. Respiratory exchange ratio (RER) was then calculated.

### 2.5 Blood sampling and biochemistry

8-week-old WT and *db/db* hamsters (n=6) were fasted for 12 hours before blood collection. Blood was collected from the retroorbital sinus into 1.5 mL microcentrifuge tubes and kept on ice. Plasma samples were isolated by centrifugation at 3000 rpm for 15–20 minutes at 4 °C and stored at -80 °C until blood biochemistry analysis. 200 μL of plasma was collected, and biochemistry was tested using the Hitachi 3500, following the manufacturer’s instructions.

The remaining serum samples were used for enzyme-linked immunosorbent assay (ELISA) to measure serum levels of leptin, fasting insulin, and HbA1c, according to the manufacturer’s protocols (BYabscience, BY-EM220624 for leptin; BY-EM221914 for insulin; BY-EM220652 for HbA1c). All samples were measured in duplicate, and the average values were used for statistical analysis (n=3).The Homeostatic Model Assessment of Insulin Resistance (HOMA-IR) index was calculated using the formula: HOMA-IR = [fasting insulin (mIU/L) × fasting glucose (mmol/L)]/22.5.

### 2.6 Blood pressure measurements

Blood pressure was measured with a BP-2000 Blood Pressure Monitor using a medium-sized hamster arm cuff. 8-week-old (n=5) and 18-week-old (n=5) WT and *db/db* hamsters were anesthetized via intraperitoneal injection of 1.25% (w/v) Avertin (Tribromoethanol + Tert-Amyl Alcohol) at a dose of 179 mg/kg body weight (≈14.3 µL/g) until immobile. Hamsters were placed in a supine position and thermally supported. Measurements were continually taken until blood pressure stabilized. For each hamster, six consecutive measurements were taken over a period of approximately 10 minutes. The average of these readings was used for analysis.

### 2.7 Proteinuria measurement

Total protein concentration in urine was quantified using the urine protein colorimetric assay kit (Sangon Biotech, D799855-0100) following the manufacturer’s protocol. Briefly, diluted urine samples from 8-week-old WT and *db/db* hamsters (n = 3), protein standards, and blanks were mixed with the provided dye reagent. After a 5-minute incubation at room temperature, the absorbance at 595 nm was measured using a SpectraMax 190 microplate reader (Molecular Devices). The protein concentration of each sample was determined by comparing its absorbance to a standard curve generated with the BSA standard. All concentrations were adjusted for the sample dilution factor.

Urinary microalbumin (mALB) was measured using a commercial ELISA kit (BYabscience, BY-EM220536) following the manufacturer’s instructions. Urinary creatinine was measured using an enzymatic assay based on the sarcosine oxidase method (BYabscience, BYSH-1204F), according to the manufacturer’s protocol. The urine albumin-to-creatinine ratio (ACR) was calculated as: ACR = urinary mALB (mg/L)/urinary creatinine (mmol/L).

### 2.8 RNA extraction and quantitative real-time RT-qPCR

Total RNA was isolated using TRIzol™ reagent (Vazyme, R411-01) according to themanufacturer’s protocol. Two micrograms of RNA were used for cDNA synthesis with reversetranscriptase (Abclonal, RK20433). Quantitative real-time PCR was performed using theLightCycler^®^ 96 System (Roche) with the Taq SYBR^®^ Green qPCR Premix (Universal) kit (Yugong Biotech, EG20117M). All steps were implemented in compliance with the manufacturer’s guidelines. The PCR cycling conditions were as follows: 30 s at 95 °C, followed by 40 cycles of 10 s at 95 °C and 30 s at 60 °C, as recommended by the manufacturer’s guidelines. Relative mRNA expression levels were calculated using the 2^−ΔΔCT^ method, with *β-actin* as the internal control. Assays were repeated three times. RT-qPCR primer sequences are provided in the **Supplementary Table 1**.

### 2.9 Western blot analysis

Tissues were collected from 8-week-old WT and *db/db* hamsters (n=3) and lysed in RIPA buffer supplemented with protease inhibitor cocktail on ice for 30 min, followed by centrifugation at 13,000 rpm for 10 min at 4 °C. Protein concentration was determined using a bicinchoninic acid (BCA) assay kit (Vazyme, E11201). A total of 30 μg of protein per sample was separated on 10% SDS–PAGE gels and transferred to PVDF membranes for immunoblotting. The following primary antibodies were used: Anti-Fatty Acid Synthase antibody (Abcam, ab128870), GAPDH Monoclonal antibody (Proteintech, 60004-1-Ig). Horseradish peroxidase (HRP)-conjugated secondary antibodies were applied (Proteintech), and signals were detected using an enhanced chemiluminescence system with SuperPico ECL Master Mix (Vazyme, E432-01).

### 2.10 Histopathology and quantification

Tissues (adipose tissue, liver, and kidney) were isolated from 8-week-old WT and *db/db* hamsters (n=3) and fixed in 4% PFA. Hematoxylin and eosin (H&E) staining, Masson’s trichrome staining, Periodic Acid-Schiff (PAS) staining, Sirius red staining, and immunofluorescence were performed by Servicebio Technology Laboratory (Wuhan, China). The images were captured using the NIS-Elements D.

Quantification of adipocyte area and size distribution based on H&E staining (n=3 animals; more than 4500 adipocytes analyzed in iBAT and more than 1000 adipocytes analyzed in iWAT and eWAT per group). The adipocyte area is calculated by ImageJ.

Quantification of liver fibrotic area and renal fibrotic area based on Masson’s trichrome staining (n=3 animals; more than 5 random fields analyzed per animal). The percentage of Masson-positive area is calculated by ImageJ.

Quantification of Immunofluorescence based on positive areas and positive cell numbers were analyzed using Saiviewer^®^ software (Servicebio, Wuhan, China) (n=3 animals per group; more than 5 random fields per animal).

### 2.11 RNA-Seq analysis

Tissues (adipose tissue, liver, and kidney) were isolated from 8-week-old WT and *db/db* hamsters (n=3), quickly frozen in liquid nitrogen, and then sent to Tenk Genomics company (Shanghai, China) for RNA-seq. All isolated RNA samples exhibited high integrity and purity, with RNA Integrity Numbers (RIN) exceeding (**Supplementary Table 2**).

Raw reads were trimmed with Trim Galore v0.6.2 to remove adapters and low-quality bases.

Clean reads were aligned to the Golden hamster (Mesocricetus auratus) reference genome (Masur2.0) using the STAR aligner (v2.7.1a) with default parameters.

Gene-level counts were obtained with featureCounts (v2.0.6). Lowly expressed genes were filtered out, retaining only those with an FPKM > 1 in at least 2 samples.

Differential expression analysis was performed using DESeq2, applying adjusted p-value < 0.05 and |log2FC| > 1 as significance thresholds.

GO enrichment analysis was conducted with AnnotationHub/AnnotationDbi, clusterProfiler, and enrichplot.

KEGG enrichment analysis was conducted with KEGGREST, clusterProfiler, and enrichplot.

### 2.12 Statistical analysis

Data are presented as the mean ± SEM. Longitudinal data (body weight, GTT, ITT, blood pressure) were analyzed by two-way ANOVA, followed by Šidák’s *post hoc* test for multiple comparisons. All other data were analyzed using unpaired Student’s t tests, with multiple comparisons corrected by the Holm-Šídák method to control the familywise error rate at α < 0.05.

For t-tests, the effect size was calculated as η² (equivalent to R² from the t-test output in Prism). For ANOVA, partial η² is reported. The 95% confidence intervals (CI) for the differences between means are reported alongside the corresponding effect sizes in the figure legends. Statistical significance is indicated as follows: *p < 0.05, **p < 0.01, ***p < 0.001; ns, not significant (exact *p*-values are reported in the figure legends and **Supplementary Table 3**).

All effect sizes were derived from, and all statistical analyses were performed using, GraphPad Prism 10 (GraphPad Software, San Diego, CA, USA). Representative results from at least three independent experiments are shown unless otherwise stated.

## 3 Results

### 3.1 Characterization of *LepR*-knockout golden hamsters

Leptin and leptin receptor (LepR) are widely expressed in mice (35). Their mRNA expression patterns are similar in both golden hamsters and mice, with *Leptin* and *LepR* primarily expressed in adipose tissue, the lung, the heart, and the liver (**Supplementary Figure 1A**).

**Figure 1:**
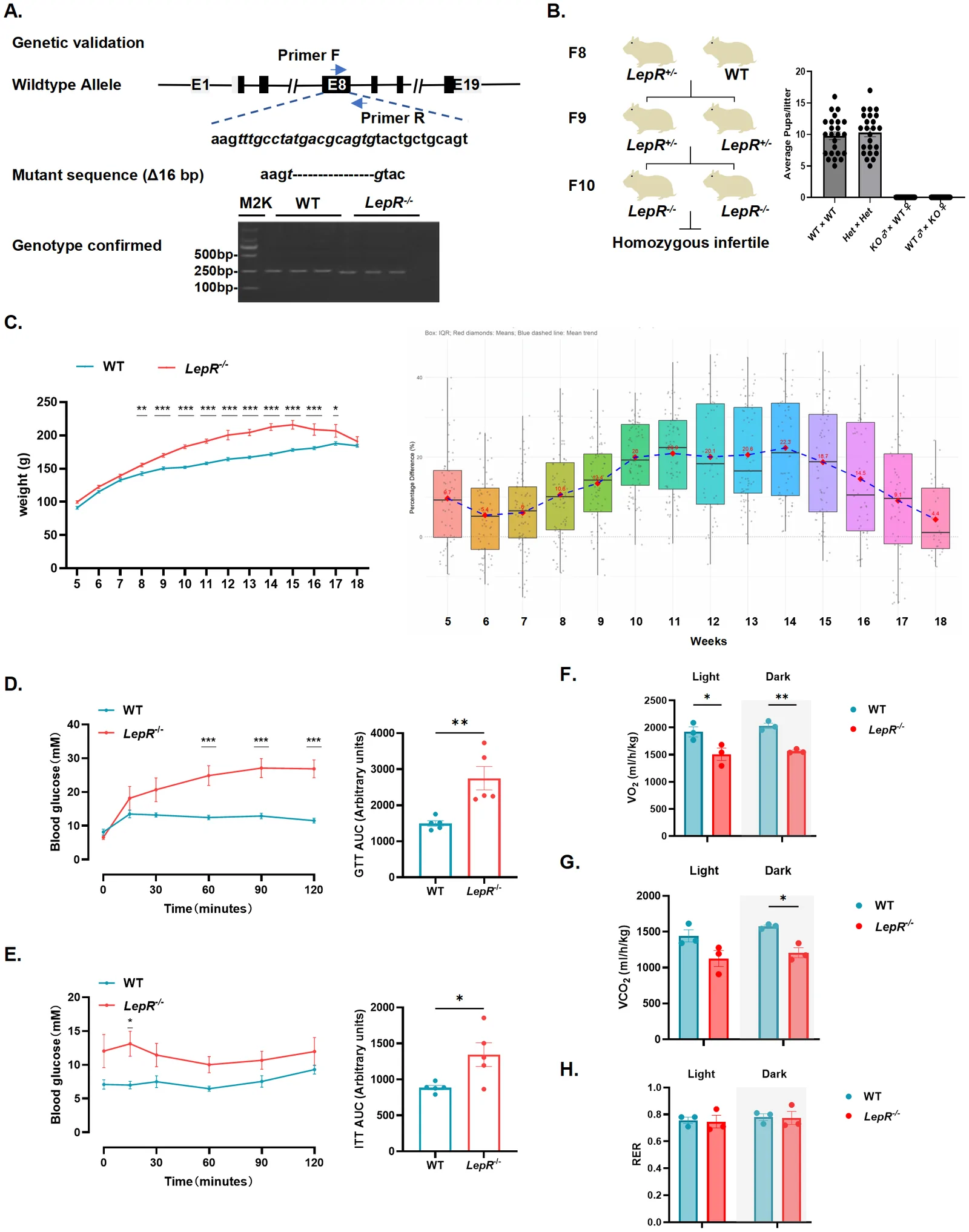
Characterization of *LepR* ablation (*db/db*) hamsters. **(A)** PCR analysis confirmed a 16-nucleotide deletion (mut16) in the *db/db* lines (n = 3). **(B)** Schematic diagram of the breeding scheme. Heterozygous (*LepR*^+^/^−^) hamsters were intercrossed to maintain the colony, as homozygous (*LepR*^−^/^−^) hamsters were infertile (Left). Litter sizes from different mating combinations (Right; n = 24). **(C)** Body weight of WT and *db/db* male golden hamsters (95% CI range: [−26.2, −20.2], *p* < 0.001, partial η² = 0.44; n ≥ 10). **(D)** Glucose tolerance test (GTT) (left; 95% CI range: [-11.2, -6.32], *p* < 0.001, partial η² = 0.52) and area under the curve (AUC) quantification (right; 95% CI [485, 2023], *p* = 0.0055, η² = 0.6387) of 8-week-old WT and *db/db* golden hamsters (n = 5). **(E)** Insulin tolerance test (ITT) (left; 95% CI range: [-5.66, -2.46], *p* < 0.001, partial η² = 0.35) and AUC quantification (right; 95% CI [73.45, 843.6], *p* = 0.0252, η² = 0.4852) of 8-week-old WT and *db/db* golden hamsters (n = 5). **(F–H)** Metabolic parameters in 8-week-old WT and *db/db* golden hamsters: oxygen consumption (VO_2_, **(F)**; Light, adjusted *p* = 0.05, Dark, adjusted *p* = 0.002), carbon dioxide production (VCO_2_, **(G)**; Dark, adjusted *p* = 0.01), and respiratory exchange ratio (RER, H). VO_2_ and VCO_2_ were measured over 24 hours, normalized to body weight, and expressed as ml/h/kg (n = 3). Data are presented as mean ± SEM. *p < 0.05, **p < 0.01, ***p < 0.001.

CRISPR/Cas9-mediated genome editing was utilized to construct a LepR-deficient golden hamster line carrying a 16-nucleotide deletion in exon 8. Genotyping confirmed the presence of the expected deletion (**Figure 1A**). Consistent with genetic knockout, quantitative analysis of five tissues (iBAT (interscapular brown adipose tissue), iWAT (inguinal white adipose tissue), eWAT (epididymal white adipose tissue), liver, and kidney) revealed that *LepR* mRNA expression was significantly downregulated in knockout golden hamsters compared with that in wild-type (WT) controls (**Supplementary Figure 1B**). Homozygous LepR*-*deficient golden hamsters were infertile, with colony maintenance depending on heterozygous intercrossing (**Figure 1B**).

### 3.2 LepR-deficient golden hamsters exhibit blood glucose levels and metabolic conditions similar to those associated with diabetes

To investigate individual development after *LepR* knockout, body weight was continuously monitored. Compared with WT controls, LepR*-*deficient male golden hamsters presented significantly greater body weights from 9 to 17 weeks; peak differences were observed at week 14 (22.3% increase compared with WT). Body weight differences gradually decreased and approached those in the WT by the end of the observation period (**Figure 1C**). Representative photographs at selected time points confirmed this trend: LepR*-*deficient golden hamsters were similar to WT controls at 3 weeks, with marked obesity by 8 weeks and pronounced central adiposity; they were comparable to WT at 19 weeks and slightly leaner than WT at 27 weeks (**Supplementary Figure 1C**). Female body weight trends were similar (**Supplementary Figure 1D**); therefore, subsequent experiments were conducted using only males. Eight-week-old golden hamsters were selected for subsequent experiments, at which point LepR*-*deficient males had already developed an obese phenotype.

GTT revealed consistently higher blood glucose levels in LepR*-*deficient golden hamsters than in WT controls during the 15–120-min period after glucose injection, despite equal baseline levels, with significant differences emerging from 60 min onward; AUC was significantly elevated in LepR*-*deficient golden hamsters. These findings indicate that LepR deficiency significantly decreased glucose metabolism (**Figure 1D**). Moreover, the ITT revealed higher fasting blood glucose levels in LepR*-*deficient golden hamsters than in WT controls. After insulin injection, blood glucose levels in LepR*-*deficient golden hamsters increased at 15 min, decreased at 30 min, and increased again at 90 min, remaining consistently higher throughout the experimental period. The AUC was significantly elevated in LepR*-*deficient golden hamsters, indicating impaired insulin sensitivity (**Figure 1E**).

To complement the functional findings, some circulating metabolic hormones and glycemic biomarkers were detected. Serum leptin, fasting insulin, and HbA1c levels were significantly elevated in LepR-deficient golden hamsters compared with those in WT controls (**Supplementary Figures 1E–G**). Consistently, the calculated HOMA-IR index was also markedly increased, indicating profound systemic insulin resistance (**Supplementary Figure 1H**).

Furthermore, TSE analysis revealed that compared with WT controls, *LepR* knockout golden hamsters had significantly decreased VO_2_ during both the light and dark periods (**Figure 1F**; **Supplementary Figure 1I**), as well as significantly decreased VCO_2_ during the dark period (**Figure 1G**, **Supplementary Figure 1J**), suggesting reduced metabolism. The RER was not significantly different, possibly because the utilization ratios of energy metabolism substrates (carbohydrates vs. lipids) were similar between the two groups (**Figure 1H**, **Supplementary Figure 1K**).

Collectively, these findings suggest that LepR-knockout golden hamsters develop metabolic syndrome characterized by obesity, glucose intolerance, insulin resistance, and decreased whole-body energy expenditure, which is consistent with the T2D phenotype. Therefore, our developed LepR*-*deficient (*db/db*) golden hamster can serve as a T2D model.

### 3.3 *LepR* knockout in golden hamsters induced dyslipidemia and inflammation

Considering the crucial role of adipose tissue in systemic metabolic regulation and its high LepR expression, we further investigated the structural and functional changes in adipose tissue.

**Figure 2:**
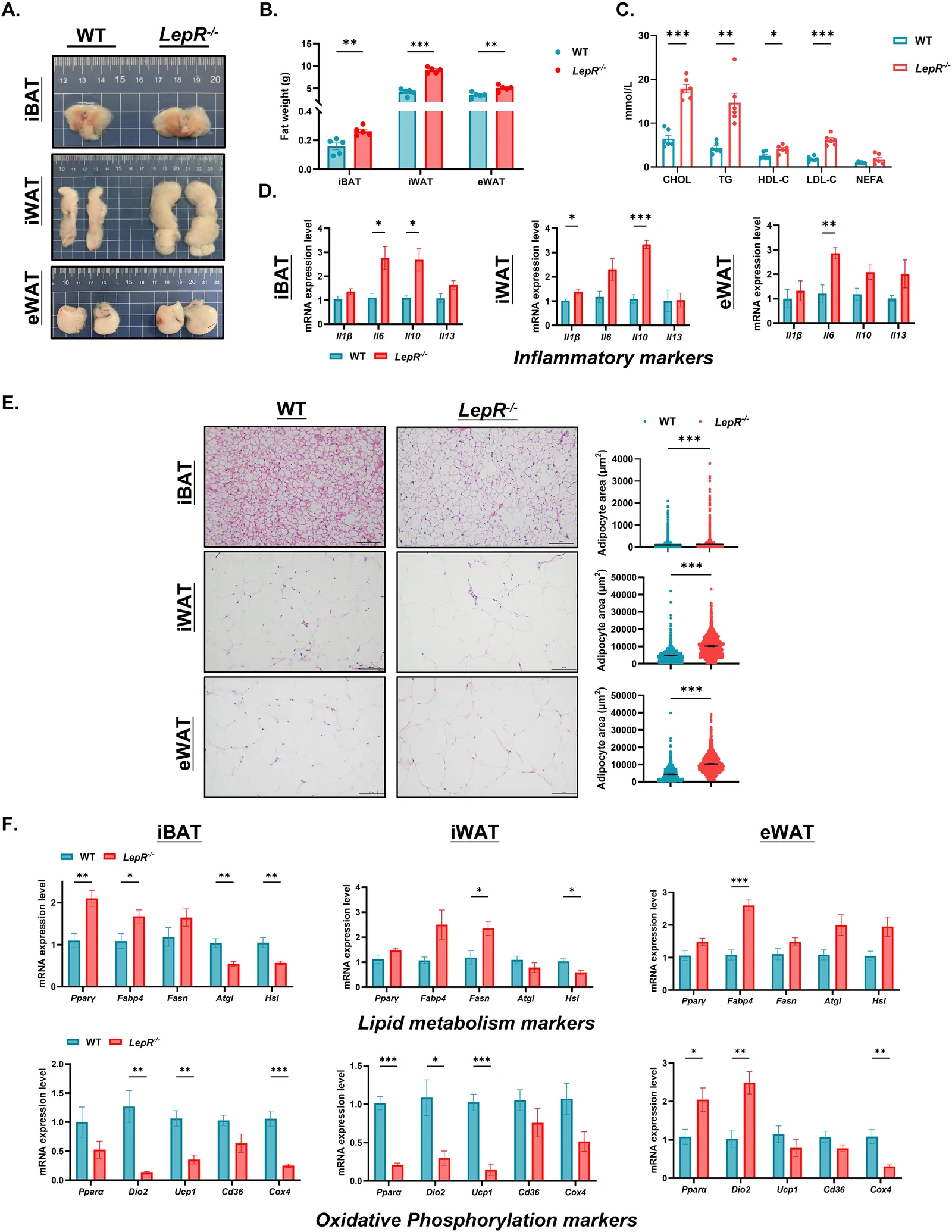
*LepR* ablation induces obesity and disrupts energy metabolism in golden hamsters. **(A)** Photographs of adipose tissues (iBAT, iWAT, and eWAT) in WT and *db/db* golden hamsters. **(B)** Adipose tissues (iBAT, iWAT, and eWAT) weight of 8-week-old WT and *db/db* golden hamsters (adjusted *p*: iBAT, *p* = 0.008, iWAT, *p* < 0.001, eWAT, *p* = 0.008; n = 5). **(C)** Serum lipid profile of 8-week-old WT and *db/db* golden hamsters (adjusted *p*: CHOL, *p* < 0.001, TG, *p* = 0.003, HDL-C, *p* = 0.02, LDL-C, *p* < 0.001; n = 6). **(D)** RT-qPCR analysis of inflammatory markers in iBAT, iWAT, and eWAT of 8-week-old WT and *db/db* golden hamsters (adjusted *p*: iBAT-*Il6*, *p* = 0.02, iBAT-*Il10*, *p* = 0.02, iWAT-*Il1β*, *p* = 0.05, iWAT-*Il10*, *p* < 0.001, eWAT-*Il6*, *p* = 0.007; n = 3). **(E)** H&E staining of iBAT, iWAT, and eWAT (left; ×20 magnification; scale bars, 100 µm) with quantification of adipocyte area (right; iBAT, 95% CI [16.50, 30.82], *p* < 0.001, η² = 0.0047; iWAT, 95% CI [5052, 5903], *p* < 0.001, η² = 0.23; eWAT, 95% CI [5574, 6297], *p* < 0.001, η² = 0.30) of 8-week-old WT and *db/db* golden hamsters (n = 3). **(F)** RT-qPCR analysis of lipid metabolism genes and oxidative phosphorylation genes in iBAT, iWAT, and eWAT of 8-week-old WT and *db/db* golden hamsters (adjusted *p*: iBAT-*Pparγ*, *p* = 0.005, *Fabp4*, *p* = 0.05, *Atgl*, *p* = 0.005, *Hsl*, *p* = 0.005, *Dio2*, *p* = 0.002, *Ucp1*, *p* = 0.001, *Cox4*, *p* < 0.001; iWAT-*Fasn*, *p* = 0.04, *Hsl*, *p* = 0.02, *Pparα*, *p* < 0.001, *Dio2*, *p* = 0.02, *Ucp1*, *p* < 0.001; eWAT-*Fabp4*, *p* < 0.001, *Pparα*, *p* = 0.05, *Dio2*, *p* = 0.005, *Cox4*, *p* = 0.004; n = 3). Total cholesterol (CHOL), triglycerides (TG), high-density lipoprotein cholesterol (HDL-C), low-density lipoprotein cholesterol (LDL-C), and non-esterified fatty acids (NEFA) Data are presented as mean ± SEM. *p < 0.05, **p < 0.01, ***p < 0.001.

To more precisely assess weight changes, we isolated adipose tissue (iBAT, iWAT, and eWAT) from WT and *db/db* golden hamsters. The weights of all three adipose depots significantly increased in the *db/db* hamsters, which was accompanied by a similar upward trend in body fat percentage (**Figures 2A, B**; **Supplementary Figure 2A**). Serum analysis revealed increased levels of total cholesterol (CHOL), triglyceride (TG), high-density lipoprotein cholesterol (HDL-C), and low-density lipoprotein cholesterol (LDL-C) in *db/db* hamsters; however, non-esterified fatty acid (NEFA) levels remained unchanged (**Figure 2C**). Collectively, these findings suggest that an increase in body fat percentage contributes to dyslipidemia development in *db/db* golden hamsters.

Furthermore, considering that visceral fat secretes inflammatory factors under these conditions, we examined the expression of the interleukin (IL) family. Although not all indicators were significantly different, the expression of *Il1β* was significantly increased in the iWAT, *Il6* was significantly increased in the iBAT and eWAT, and *Il10* was significantly increased in the iBAT and iWAT of *db/db* golden hamsters compared to WT controls, indicating substantial inflammatory responses across adipose tissues (**Figure 2D**). To further characterize the inflammatory status of adipose tissue, we performed immunofluorescence staining for CD68, a marker of macrophages. Notably, the CD68-positive area was significantly increased in the iWAT and eWAT of the *db/db* golden hamsters compared to WT controls, indicating active macrophage infiltration in these depots. In contrast, the CD68-positive area did not significantly change in the iBAT, suggesting a tissue-specific pattern of macrophage recruitment in response to metabolic stress (**Supplementary Figure 2B**).

In addition, to examine the cellular basis of the metabolic phenotype, we analyzed adipose tissue pathology and gene expression profiles. Morphometric analysis revealed an increased average adipocyte area in the adipose tissue of *db/db* golden hamsters. In particular, in iBAT, lipid droplets tended to fuse into larger structures, with a greater proportion of droplets exceeding 2,000 μm^2^. In iWAT and eWAT, the proportion of hypertrophic cells exceeding 30,000 μm^2^ markedly increased, with a corresponding decrease in the number of small droplets (**Figure 2E**; **Supplementary Figure 2C**).Consistent with these morphological changes, we noted tissue-specific alterations in gene expression. In iBAT and iWAT, lipogenic genes were upregulated (e.g. *Pparγ* and *Fasn*), whereas genes involved in lipolysis (e.g. *Atgl* and *Hsl*) and oxidative phosphorylation (e.g. *Pparα* and *Dio2)* were downregulated. In eWAT, a distinct pattern emerged, characterized by significant upregulation of *Pparα* and *Dio2* (**Figure 2F**).

Collectively, the findings in *db/db* golden hamsters revealed increased lipid deposition and impaired lipolysis, resulting in dyslipidemia similar to that observed in patients with T2D, which is consistent with the findings in *db/db* mice (36, 37).

### 3.4 *LepR* knockout induced a diabetes-associated gene expression signature in adipose tissue

Next, we subjected iBAT, iWAT, and eWAT from *db/db* and WT golden hamsters to RNA-seq to elucidate the molecular mechanisms underlying *LepR*-regulated lipid metabolism. Principal component analysis (PCA) and sample correlation analysis demonstrated a distinguishable clustering pattern between genotypes, with biological replicates clustering closely within each group, indicating consistent transcriptomic differences between WT and *db/db* samples (**Supplementary Figures 3A–C**).

**Figure 3:**
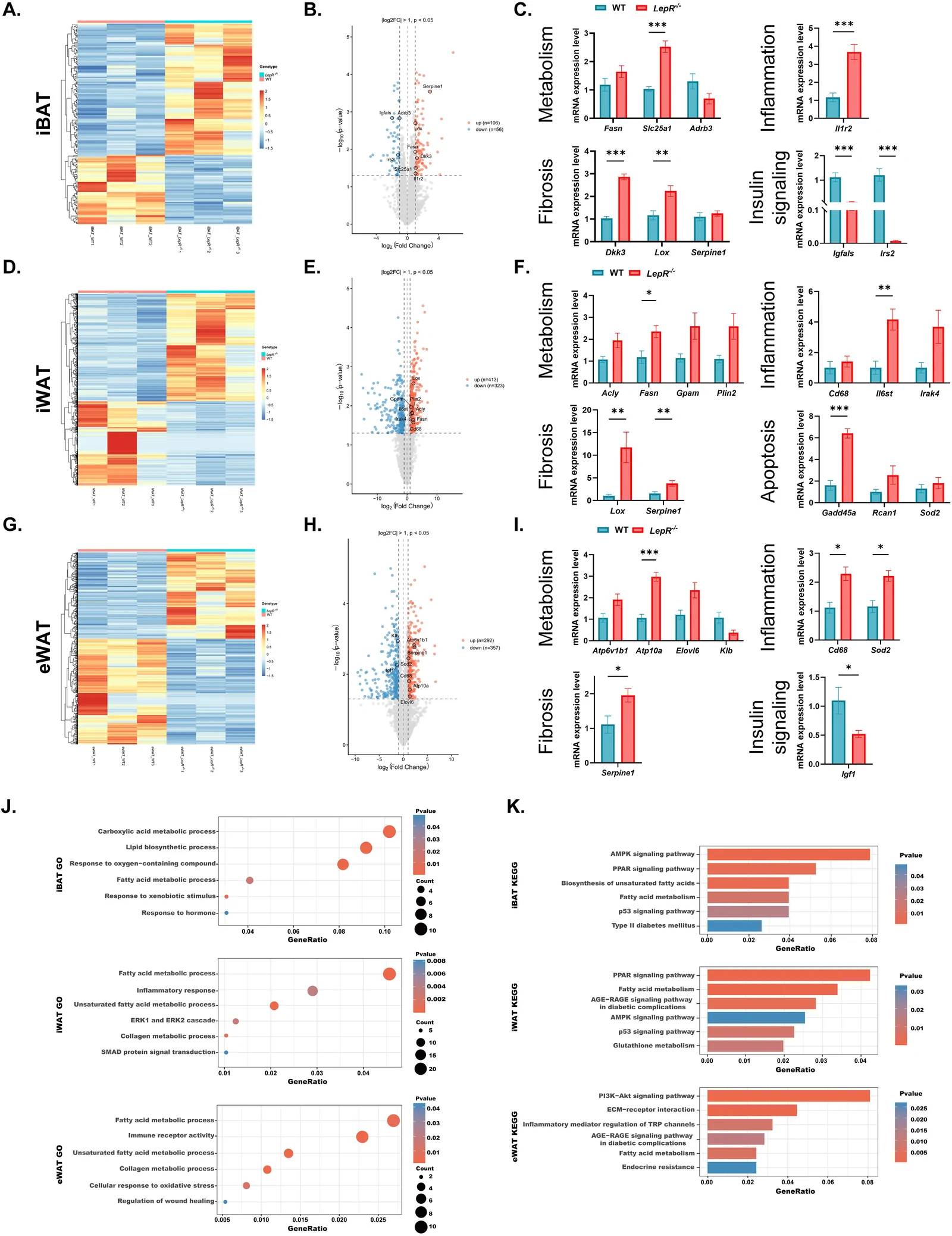
Transcriptomic alterations in adipose tissues of *db/db* golden hamsters. **(A–C)** Interscapular brown adipose tissue (iBAT): **(A)** Heatmap of differentially expressed genes in iBAT between *db/db* and WT golden hamsters (8-week-old; n = 3). **(B)** Volcano plot of differentially expressed genes (DEGs). **(C)** RT-qPCR results of selected DEGs (n = 3). **(D–F)** Inguinal white adipose tissue (iWAT): **(D)** Heatmap of differentially expressed genes in iWAT between *db/db* and WT golden hamsters (8-week-old; n = 3). **(E)** Volcano plot of DEGs. **(F)** RT-qPCR results of selected DEGs (n = 3). **(G–I)** Epididymal white adipose tissue (eWAT): **(G)** Heatmap of differentially expressed genes in eWAT between *db/db* and WT golden hamsters (8-week-old; n = 3). **(H)** Volcano plot of DEGs. **(I)** RT-qPCR results of selected DEGs (n = 3). **(J)** GO enrichment analysis of iBAT, iWAT, and eWAT DEGs. Dots represent significantly enriched terms (*p* < 0.05). **(K)** KEGG pathway enrichment analysis of iBAT, iWAT, and eWAT DEGs. Bars represent significantly enriched pathways (*p* < 0.05). Data are presented as mean ± SEM. *p < 0.05, **p < 0.01, ***p < 0.001, exact adjusted *p*-values are provided in **Supplementary Table 3**.

In iBAT, 106 genes were upregulated, whereas 56 were downregulated (>2-fold change; **Figures 3A, B**). We selected eight candidate genes associated with metabolism (e.g., *Adrb3*), inflammation (*Il1r2*), fibrosis (e.g., *Dkk2*), and insulin signaling (e.g., *Igfals*) for validation via RT-qPCR, confirming RNA-seq results (**Figure 3C**, **Supplementary Figure 3D**). In iWAT, 413 genes were upregulated, whereas 323 were downregulated (>2-fold change; **Figures 3D, E**). Eleven candidate genes associated with metabolism (e.g., *Acly*), inflammation (e.g., *Cd68*), fibrosis (e.g., *Lox*), and apoptosis (e.g., *Gadd45a*) were validated via RT-qPCR (**Figure 3F**, **Supplementary Figure 3E**). In eWAT, 292 genes were upregulated, whereas 357 were downregulated (>2-fold change; **Figures 3G, H**). Eight selected genes associated with metabolism (e.g., *Atp6v1b1*), inflammation (e.g., *Cd68*), fibrosis (*Serpine1*), and insulin signaling (*Igf1*) were subjected to RT-qPCR validation, confirming consistency with the transcriptomic data (**Figure 3I**, **Supplementary Figure 3F**).

Gene Ontology (GO) analysis of transcriptomic profiles revealed that all three adipose tissue types were enriched for the biological process “Fatty acid metabolic process,” as well as for the pathways associated with inflammation. Notably, candidate genes such as *Fasn*, *Irs2*, and *Serpine1* were validated at the mRNA level and were noted to be implicated in these pathways (**Figure 3J**). KEGG analysis of differentially expressed genes (DEGs) revealed that “Fatty acid metabolism” and the pathways associated with diabetes (e.g., “Type II diabetes mellitus” and “AGE-RAGE signaling pathway in diabetic complications”) were significantly enriched across all three adipose tissue types. Accordingly, candidate genes such as *Fasn*, *Gadd45a*, and *Igf1* were validated at the mRNA level and were noted to be associated with these pathways (**Figure 3K**).

Differential expression and enrichment analyses revealed that *LepR* knockout affected multiple diabetes-related pathways by significantly changing the expression of genes involved in metabolism, fibrosis, inflammation, and apoptosis.

### 3.5 LepR deficiency induced metabolic dysfunction-associated steatotic liver disease (MASLD) with early fibrosis in golden hamsters

Considering that T2D is a systemic disease, we further investigated whether *LepR* knockout affects other tissues in golden hamsters during the early stages. Given the elevated LDL-C and TG levels and the key role of the liver in lipid metabolism under obesity and insulin resistance conditions, we performed a comprehensive liver phenotypic analysis.

**Figure 4:**
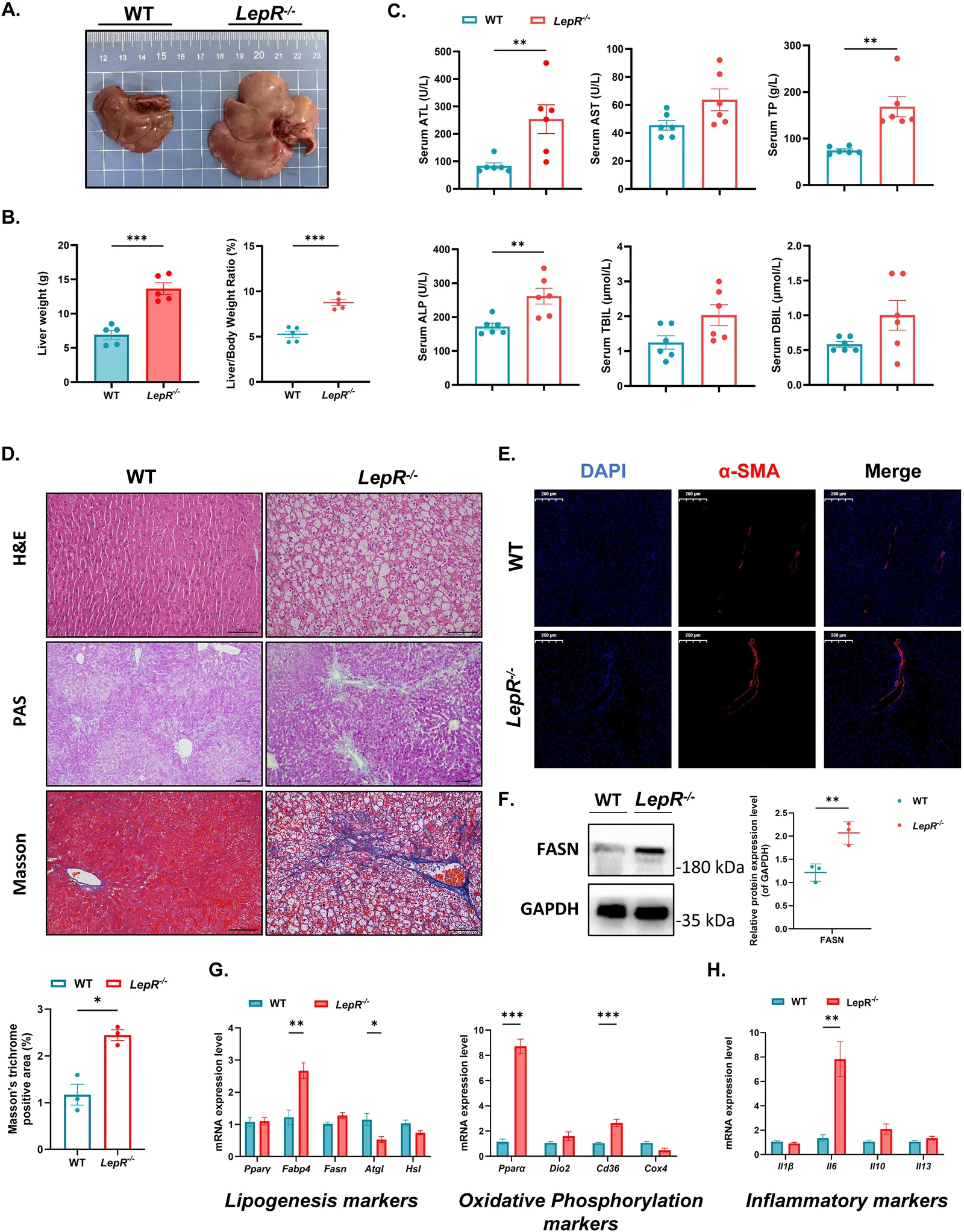
LepR deficiency induces impaired liver function with a mild fibrotic phenotype in golden hamsters. **(A)** Photographs of the liver in 8-week-old WT and *db/db* golden hamsters. **(B)** Liver weight (left; 95% CI [4.302, 9.182], *p* = 0.0002, η² = 0.8353) and liver to body weight ratio (right; 95% CI [2.386, 4.618], *p* < 0.001, η² = 0.8675) of 8-week-old WT and *db/db* golden hamsters (n = 5). **(C)** Serum liver function profile of 8-week-old WT and *db/db* golden hamsters (ATL, 95% CI [50.76, 289.2], *p* = 0.0099, η² = 0.5023; TP, 95% CI [45.48, 142.4], *p* = 0.0015, η² = 0.6511; ALP, 95% CI [32.93, 146.4], *p* = 0.0055, η² = 0.5535; n = 6). **(D)** Histological staining of liver sections (top: H&E and Masson’s trichrome: ×20 magnification; PAS: ×10 magnification. Scale bars, 100 µm for all panels) and quantification of fibrotic area based on Masson’s trichrome staining (bottom; 95% CI [0.4765, 2.074], *p* = 0.01, η² = 0.8970) (8-week-old WT and *db/db* golden hamsters, n = 3). **(E)** Representative images of α-SMA (red) staining in liver from WT and *db/db* golden hamsters (×4 magnification; scale bars, 250 µm). Nuclei were counterstained with DAPI (blue) (8-week-old WT and *db/db* golden hamsters, n=3). **(F)** Western blot of FASN in the liver of 8-week-old WT and *db/db* golden hamsters, GAPDH as a reference gene (95% CI [0.3526, 1.354], *p* = 0.01, η² = 0.8613; n = 3). **(G)** RT-qPCR analysis of lipid metabolism genes and oxidative phosphorylation genes in the liver of 8-week-old WT and *db/db* golden hamsters (adjusted *p*: *Fabp4*, *p* = 0.003, *Atgl*, *p* = 0.04, *Pparα*, *p* < 0.001, *Cd36*, *p* < 0.001; n = 3). **(H)** RT-qPCR analysis of inflammatory markers in the liver of 8-week-old WT and *db/db* golden hamsters (*Il6* adjusted *p* = 0.002; n = 3). ALT, Alanine aminotransferase; AST, aspartate aminotransferase; TP, total protein; ALP, alkaline phosphatase; TBIL, total bilirubin; DBIL, direct bilirubin. Data are presented as mean ± SEM. *p < 0.05, **p < 0.01, ***p < 0.001.

First, the liver weight and liver-to-body weight ratio were significantly increased in *db/db* golden hamsters than in WT controls (**Figures 4A, B**). Furthermore, *db/db* golden hamsters exhibited elevated serum alanine aminotransferase (ALT) and aspartate aminotransferase (AST) levels, indicating liver cell damage. Furthermore, hepatic protein synthesis was impaired, accompanied by increased total protein (TP) levels. In addition, bilirubin metabolism was affected, resulting in cholestasis, as evidenced by elevated alkaline phosphatase (ALP), total bilirubin (TBIL), and direct bilirubin (DBIL) levels (**Figure 4C**).

Subsequent histopathological analysis revealed hepatocyte ballooning, a hallmark of MASLD. PAS staining revealed glycogen deposition in hepatocytes, consistent with early metabolic derangement in this model. Sirius red staining showed positive signals mainly confined to vascular walls, whereas Masson’s trichrome staining revealed a significant increase in fibrotic areas within hepatic lobules compared to WT controls, as confirmed by quantitative analysis of the positive area (**Figure 4D**, **Supplementary Figure 4A**).

To assess hepatic stellate cell activation, we performed immunofluorescence staining for α-SMA. In *db/db* golden hamsters, α-SMA-positive signals extended from the portal areas into the adjacent hepatic parenchyma, a pattern not observed in WT controls (**Figure 4E**). This localized distribution pattern indicates the early onset of metabolic fibrotic remodeling in the *db/db* liver.

Given that FASN is a crucial enzyme involved in hepatic lipogenesis and is upregulated in association with insulin resistance (38), we further validated the changes in FASN protein levels. We observed that FASN levels were increased in the liver of *db/db* golden hamsters (**Figure 4F**). Transcriptional analysis further revealed that lipogenesis-related genes were significantly upregulated, whereas those related to lipolysis were downregulated. Moreover, the expression of genes related to oxidative phosphorylation was upregulated, whereas *Cox4* expression was downregulated (**Figure 4G**).

Furthermore, we examined the IL family in the liver. Among the tested cytokines, *Il6* expression was significantly elevated in *db/db* hamsters compared to WT controls, whereas *Il10* and *Il13* showed increasing trends that did not reach statistical significance. *Il1β* expression slightly but non-significantly decreased (**Figure 4H**). Immunofluorescence staining for CD68 revealed a trend toward an increased positive area in the liver of *db/db* hamsters compared with WT controls, although this difference did not reach statistical significance (**Supplementary Figure 4B**).

Collectively, our findings suggest that *LepR* knockout in *db/db* golden hamsters impaired liver function, resulting in MASLD with mild fibrosis, features resembling key pathological aspects of MASLD, potentially involving disrupted lipid droplet homeostasis and early fibrotic remodeling pathways.

To determine the mechanisms by which LepR deficiency drives hepatic pathology, we subjected liver tissue from *db/db* golden hamsters and WT controls to RNA-seq. PCA and sample correlation analysis demonstrated a distinguishable clustering pattern between genotypes, with biological replicates clustering closely within each group, indicating consistent transcriptomic differences between WT and *db/db* samples (**Supplementary Figure 5A**).

**Figure 5:**
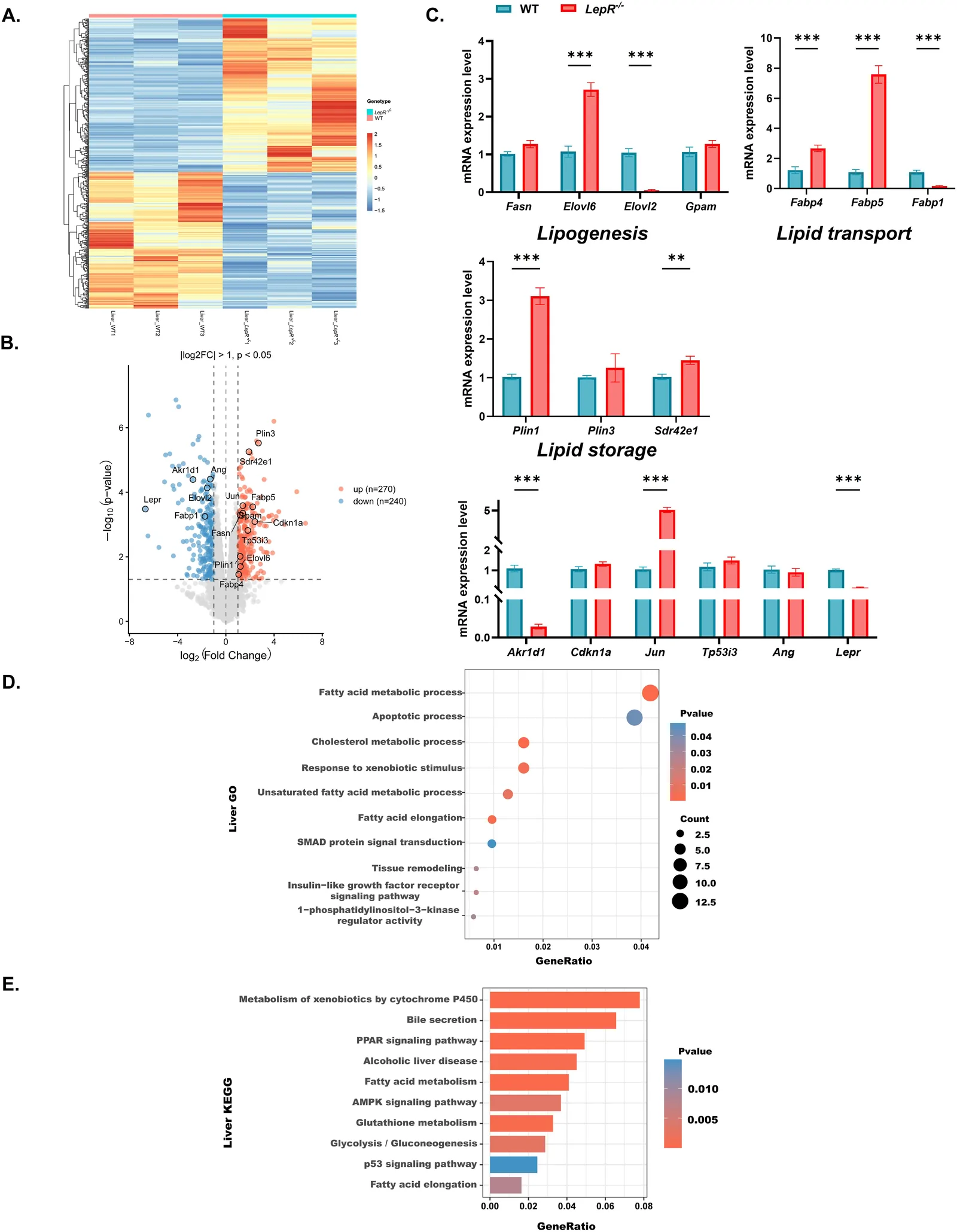
Transcriptomic alterations in the liver of *db/db* golden hamsters. **(A)** Heatmap of gene changes in *db/db* vs. WT liver (8-week-old, n = 3). **(B)** Volcano plot of differentially expressed genes (DEGs). **(C)** RT-qPCR results of selected DEGs (n = 3) **(D)** GO enrichment analysis of liver DEGs. Dots represent significantly enriched terms (*p* < 0.05). **(E)** KEGG pathway enrichment analysis of liver DEGs. Bars represent significantly enriched pathways (*p* < 0.05). Data are presented as mean ± SEM. **p < 0.01, ***p < 0.001, exact adjusted *p*-values are provided in **Supplementary Table 3**.

We observed that 270 genes were upregulated and 240 genes were downregulated in the liver (>2-fold change; **Figures 5A, B**). We focused on 16 candidate genes associated with lipid metabolism, including lipogenesis (e.g., *Fasn*), lipid transport (e.g., *Fabp4*), and lipid storage (e.g., *Plin1*). These candidate genes are also associated with bile secretion (*Akr1d1*), cell fate decisions (e.g., *Cdkn1a*), and circulatory function (*Ang*). The RNA-seq results were validated via RT-qPCR (**Figure 5C**, **Supplementary Figure 5B**).

GO analysis revealed that DEGs were significantly enriched in biological processes such as “Fatty acid metabolic process” and “Apoptotic process” in the liver. Notably, the candidate genes *Elovl2*, *Elovl6*, and *Fasn* were validated at the mRNA level and were noted to be associated with these pathways (**Figure 5D**). Furthermore, KEGG analysis revealed significant enrichment for biological processes, including “Alcoholic liver disease” and “p53 signaling pathway” in the liver. Accordingly, candidate genes such as *Fabp1*, *Cdkn1a*, and *Tp53i3* were validated at the mRNA level and were noted to be associated with these pathways (**Figure 5E**).

Collectively, RNA-seq analysis revealed that LepR deficiency induced lipid dysregulation and fibrotic stress in *db/db* golden hamsters, with DEGs enriched in biological processes associated with steatosis and fibrosis. Furthermore, our results revealed that LepR deficiency plays a key role in key pathways associated with liver insulin resistance (e.g., “1-phosphatidylinositol 3-kinase regulator activity”).

### 3.6 A metabolism–inflammation–tissue remodeling axis underlies renal injury in *db/db* golden hamsters

**Figure 6:**
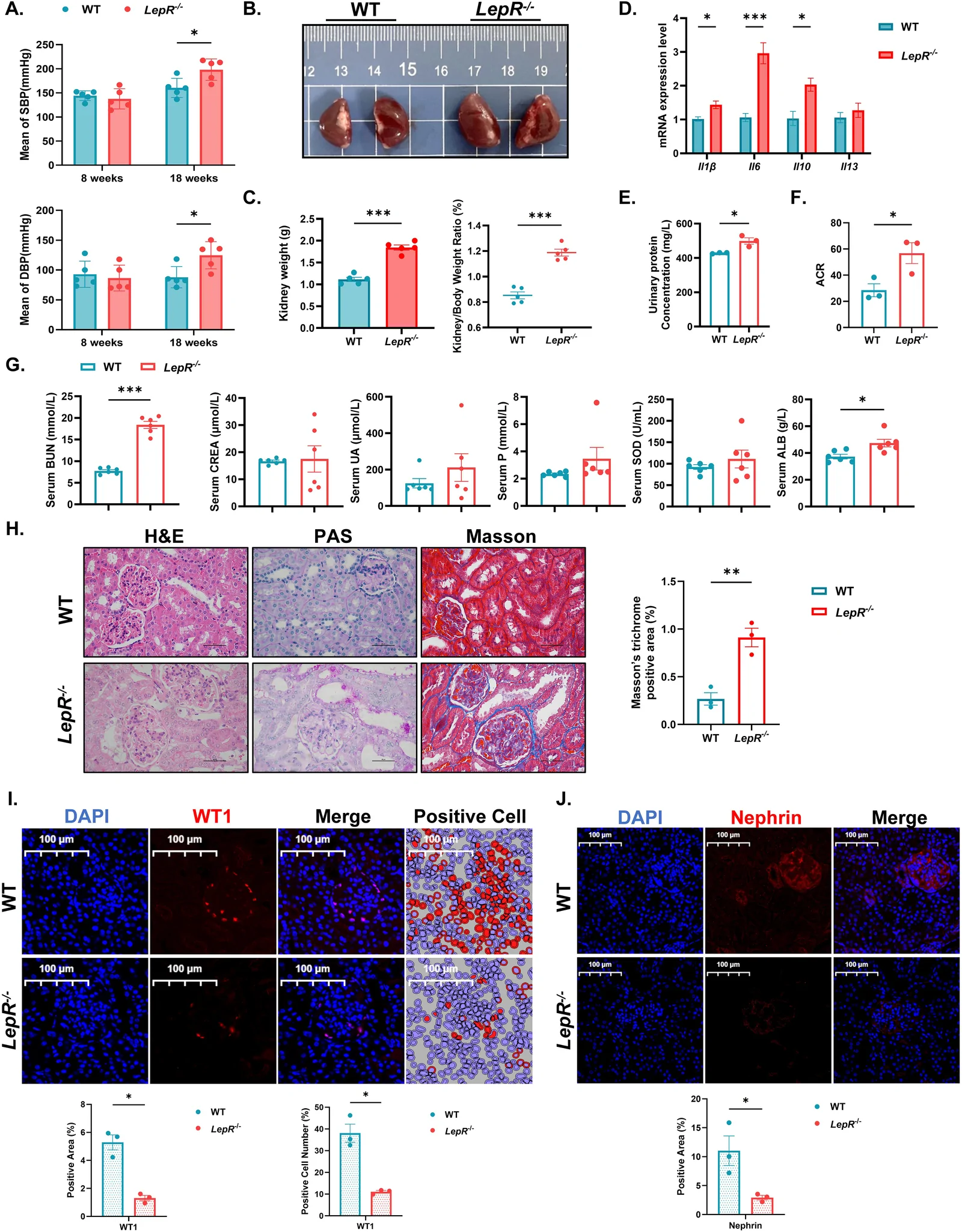
LepR deficiency induces hypertension and renal injury in golden hamsters. **(A)** Systolic (top) and diastolic (bottom) blood pressure measurements in WT and *db/db* golden hamsters (18-weeks-SBP, adjusted *p* = 0.01, 18-weeks-DBP, adjusted *p* = 0.03; 8-weeks-old, n = 5; 18-weeks-old, n = 5). **(B)** Photographs of the kidneys of 8-week-old WT and *db/db* golden hamsters. **(C)** Kidney weight (left; 95% CI [0.5573, 0.9029], *p* < 0.001, η² = 0.9223) and kidney to body weight ratio (right; 95% CI [0.2476, 0.4244], *p* < 0.001, η² = 0.9058) of 8-week-old WT and *db/db* golden hamsters (kidney weight, n = 5; kidney to body weight ratio, n = 5). **(D)** RT-qPCR analysis of inflammatory markers in the kidney of 8-week-old WT and *db/db* golden hamsters (adjusted *p*: Il1β, *p* = 0.01, *Il6*, *p* < 0.001, *Il10*, *p* = 0.01; n = 3). **(E)** Urine protein detection of 8-week-old WT and *db/db* golden hamsters (95% CI [18.28, 122.2], *p* = 0.02, η² = 0.7788; n = 3). **(F)** Urinary albumin-to-creatinine ratio (ACR) in WT and *db/db* golden hamsters (95% CI [0.2703, 56.41], *p* = 0.05, η² = 0.7335; n = 3). **(G)** Serum renal function profile of 8-week-old WT and *db/db* golden hamsters (BUN,95% CI [8.921, 12.36], *p* < 0.001, η² = 0.9806; ALB, 95% CI [2.687, 17.55], *p* = 0.01, η² = 0.4793; n = 6). **(H)** Histological staining of kidney sections: glomeruli (H&E, PAS, Masson’s trichrome; ×40 magnification; scale bars, 50 µm). Quantification of renal fibrotic area based on Masson’s trichrome staining (95% CI [0.2987, 0.9880], *p* = 0.008, η² = 0.8968; 8-week-old WT and *db/db* golden hamsters, n = 3). **(I)** Representative images of WT1 (red) staining in renal sections from WT and *db/db* golden hamsters (×10 magnification; scale bars, 100 µm). Nuclei were counterstained with DAPI (blue). Quantification of WT1-positive area (95% CI [-6.009, -1.948], *p* = 0.01, η² = 0.9517) and WT1-positive cell number (95% CI [-44.47, -9.500], *p* = 0.02, η² = 0.9515; 8-week-old WT and *db/db* golden hamsters, n = 3). **(J)** Representative images of Nephrin (red) staining in renal sections from WT and *db/db* golden hamsters (×10 magnification; scale bars, 100 µm). Nuclei were counterstained with DAPI (blue). Quantification of Nephrin-positive area (95% CI [-15.30, -0.9206], *p* = 0.04, η² = 0.7103; 8-week-old WT and *db/db* golden hamsters, n = 3). Blood urea nitrogen (BUN), creatinine (CREA), uric acid (UA), phosphorus (P), superoxide dismutase (SOD), and albumin (ALB) Data are presented as mean ± SEM. *p < 0.05, **p < 0.01, ***p < 0.001.

Notably, blood pressure monitoring revealed hypertension in *db/db* golden hamsters (**Figure 6A**). This feature is not present in typical *db/db* mice but is a characteristic of DN. Subsequently, we further explored their renal phenotype. Kidney weight and the kidney-to-body weight ratio were significantly increased in *db/db* golden hamsters compared with WT controls (**Figures 6B, C**). Considering that sustained hypertension can result in endothelial damage and inflammation, we measured the expression of IL family members in the kidney. Notably, *Il1β, Il6*, *Il10*, and *Il13* expression was upregulated in *db/db* hamsters, with *Il1β, Il6*, *Il10* exhibiting a statistically significant increase (**Figure 6D**). Immunofluorescence staining for CD68 revealed a trend toward an increased positive area in the kidneys of *db/db* hamsters compared with those of WT controls; this difference did not reach statistical significance (**Supplementary Figure 6A**).

Furthermore, urinalysis revealed increased urinary protein excretion, indicating impaired glomerular filtration barrier function (**Figure 6E**). To evaluate glomerular integrity, we also measured urinary microalbumin (mALB) and urinary creatinine levels and then calculated the albumin-to-creatinine ratio (ACR). Both mALB levels and ACR were significantly elevated in *db/db* golden hamsters compared to WT controls (**Figure 6F**, **Supplementary Figure 6B**), confirming a defective glomerular filtration function.

Blood biochemistry analysis revealed a marked increase in blood urea nitrogen (BUN) and albumin (ALB), an early marker of DN. However, there were no significant changes in the levels of creatinine (CREA), uric acid (UA), phosphorus (P), or superoxide dismutase (SOD). Although not all markers exhibited statistically significant changes, our findings further suggest impaired renal excretory function, possibly accompanied by metabolic imbalance or early kidney damage (**Figure 6G**).

Considering the abovementioned tests indicated kidney injury, we subsequently performed pathological analysis. The results revealed changes in glomerular basement membrane thickness (**Figure 6H**) and shedding of renal tubular epithelial cells (**Supplementary Figure 6C**). Masson’s trichrome staining revealed renal fibrosis, an early feature of DN. To further characterize the molecular alterations underlying renal injury, we performed immunofluorescence staining for key markers of DN. The WT1-positive area and positive cell percentage were significantly decreased in *db/db* kidneys compared to WT controls (**Figure 6I**), indicating podocyte loss. The Nephrin-positive area was also significantly reduced, suggesting disruption of the slit diaphragm (**Figure 6J**). Collagen I and α-SMA positive areas were significantly increased (**Supplementary Figure 7**), reflecting active myofibroblast activation and matrix deposition, whereas the fibronectin-positive area did no significantly change.

Collectively, these findings suggested that LepR deficiency results in early-stage renal injury, including inflammation, filtration barrier damage, and structural changes consistent with early DN.To investigate the specific molecular mechanisms underlying DN in *db/db* golden hamsters, we subjected kidney tissues from *db/db* and WT golden hamsters to RNA-seq. PCA and sample correlation analysis demonstrated a distinguishable clustering pattern between genotypes, with biological replicates clustering closely within each group, indicating consistent transcriptomic differences between WT and *db/db* samples (**Supplementary Figure 8A**).

**Figure 7:**
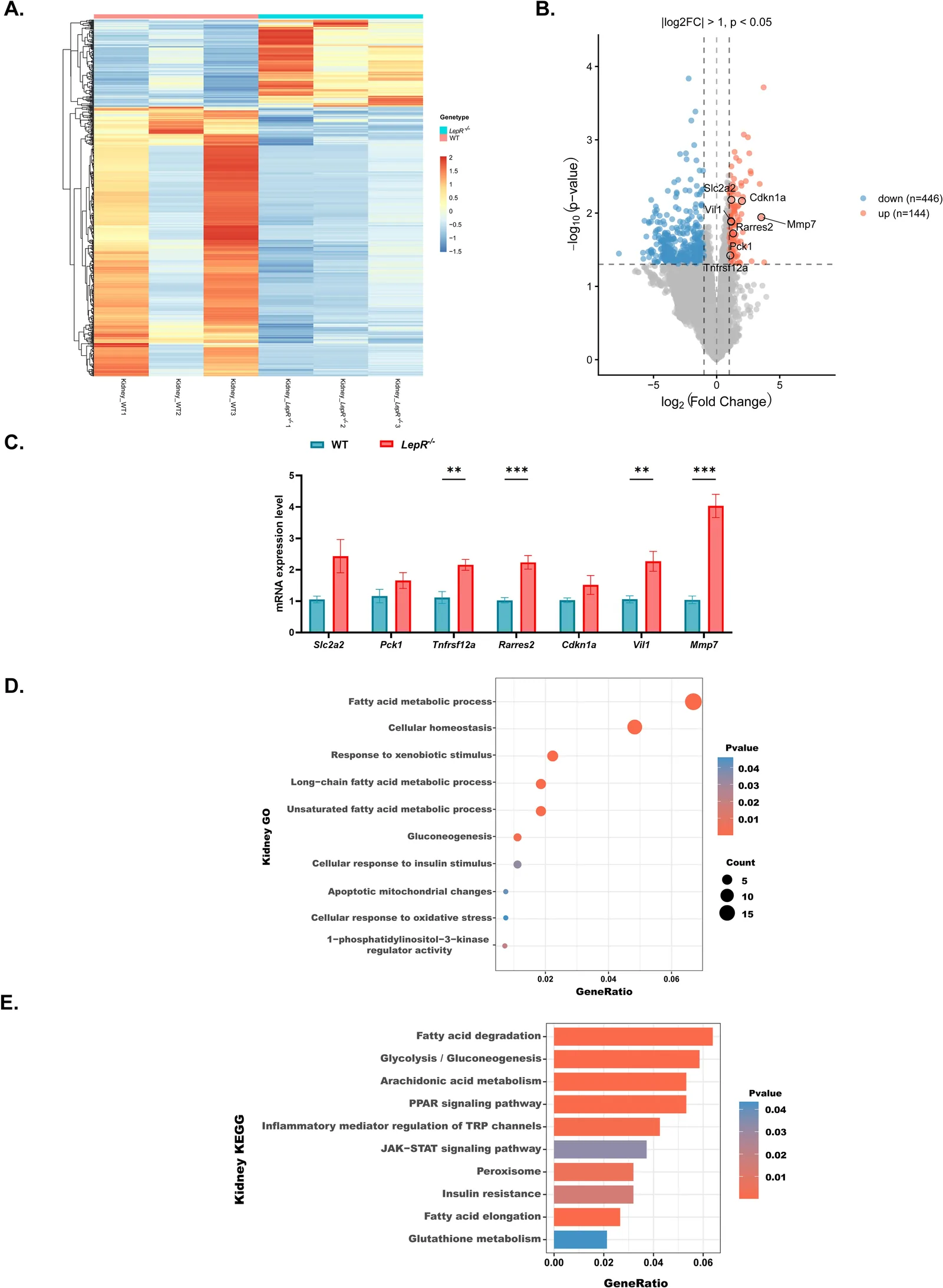
Transcriptomic alterations in the kidney of *db/db* golden hamsters. **(A)** Heatmap of gene changes in *db/db* vs. WT kidney (8-week-old, n = 3). **(B)** Volcano plot of differentially expressed genes (DEGs). **(C)** RT-qPCR results of selected DEGs (n = 3). **(D)** GO enrichment analysis of kidney DEGs. Dots represent significantly enriched terms (*p* < 0.05). **(E)** KEGG pathway enrichment analysis of kidney DEGs. Bars represent significantly enriched pathways (*p* < 0.05). Data are presented as mean ± SEM. *p < 0.05, **p < 0.01, ***p < 0.001, exact adjusted *p*-values are provided in **Supplementary Table 3**.

In total, 144 genes were upregulated and 446 genes were downregulated in the kidney (>2-fold change; **Figures 7A, B**). Next, we validated the RNA-seq results of nine candidate genes associated with functions such as lipid metabolism (*Slc2a2*), gluconeogenesis (*Pck1*), inflammation (e.g., *Tnfrsf12a*), cell decision (*Cdkn1a*), and nephropathy (e.g., *Vil1*) via RT-*q*PCR (**Figure 7C**, **Supplementary Figure 8B**). GO analysis revealed that renal DEGs were significantly enriched in biological processes such as “Fatty acid metabolic process” and “Gluconeogenesis”. Candidate genes such as *Pck1* were validated at the mRNA level and were noted to be functionally associated with these pathways (**Figure 7D**). Furthermore, KEGG analysis revealed significant enrichment in pathways such as “JAK-STAT signaling pathway” and “Insulin resistance” in the kidney. Consistent with these findings, candidate genes such as *Cdkn1a* and *Slc2a2* were validated at the mRNA level and were noted to be implicated in these pathways (**Figure 7E**).

Collectively, RNA-seq analysis revealed that *LepR* knockout induces a metabolism–inflammation–tissue remodeling axis that serves as the key molecular foundation for fibrotic progression in DN in *db/db* golden hamsters. This pathogenic axis is initiated owing to disruptions in gluconeogenesis and lipid metabolism.

### 3.7 Integrative multi-species analysis identified conserved transcriptional targets in *db/db* golden hamsters and human DN

**Figure 8:**
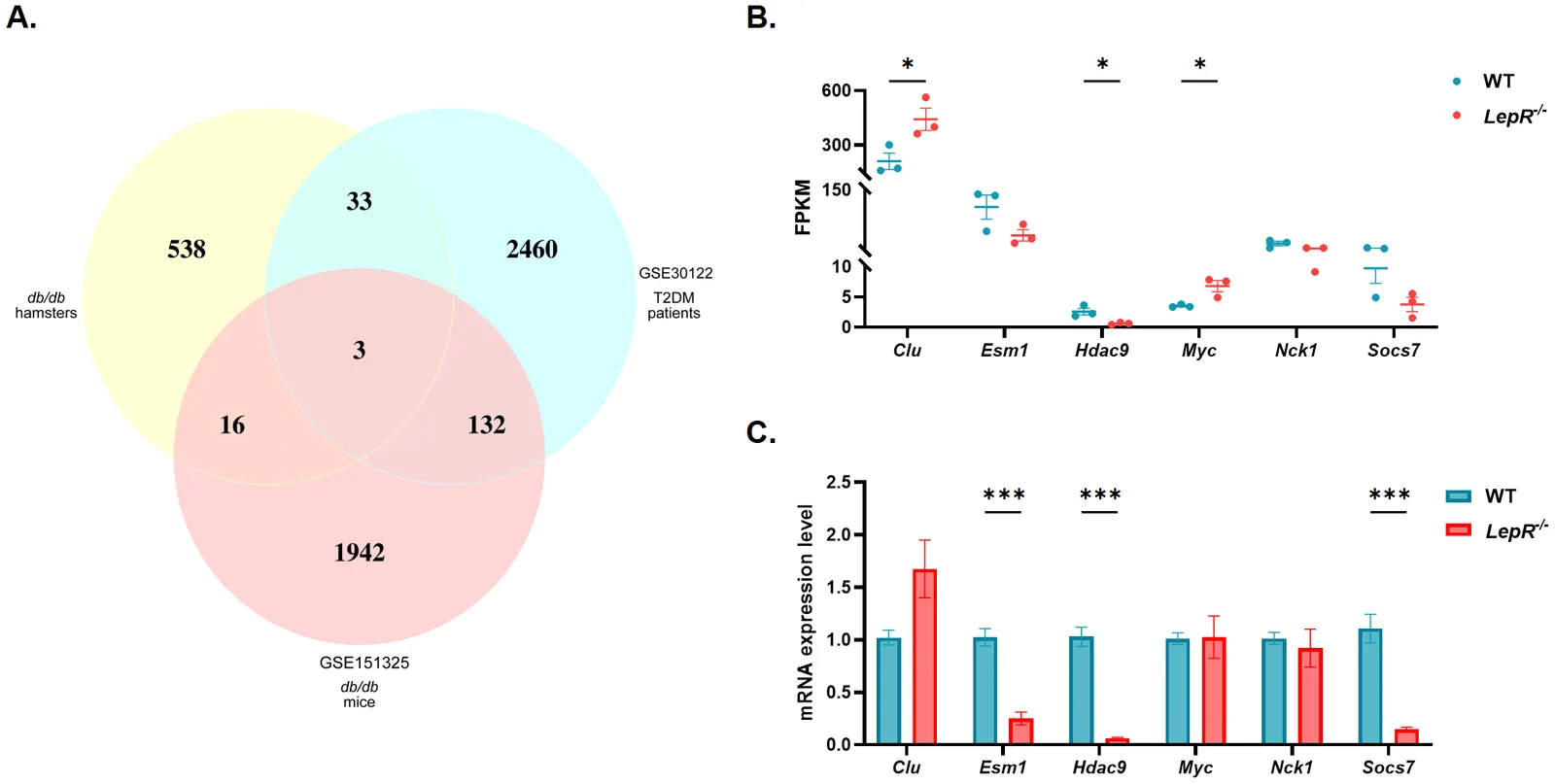
Compared the publicly available DN datasets with *db/db* golden hamsters. **(A)** Venn diagram of the common regulated gene in kidney tissue of *db/db* golden hamsters, T2D patients and *db/db* mice. **(B)** FPKM of selected DEGs in the kidney, which were commonly regulated in *db/db* golden hamsters and T2D patients. **(C)** Validation of candidate genes by RT-qPCR (n = 3). Data are presented as mean ± SEM. *p < 0.05, **p < 0.01, ***p < 0.001, exact adjusted *p*-values are provided in **Supplementary Table 3**.

To further identify the targets and molecular mechanisms underlying DN, we analyzed two publicly available microarray datasets (GSE30122 for patients with T2D and GSE151325 for *db/db* mice) and an RNA-seq dataset from the kidneys of our *db/db* golden hamsters. Compared with DEGs, we filtered for genes commonly regulated across these three datasets and generated a Venn diagram of the intersection. We identified 33 shared genes between patients with T2D and *db/db* golden hamsters (**Figure 8A**). Among these, we selected 6 candidate genes (e.g., *Clu*) with established roles in DN pathogenesis. RT-qPCR was used to validate the mRNA expression of these 6 genes in the kidneys of *db/db* golden hamsters (**Figures 8B, C**). This integrated cross-species approach successfully identified conserved key genes, strengthening the translational relevance of *db/db* golden hamsters for DN research.

## 4 Discussion

In this study, we metabolically, histopathologically, and transcriptomically characterized a LepR-deficient (*db/db*) golden hamster model. The model exhibited progressive obesity, severe hyperglycemia, insulin resistance, dyslipidemia, spontaneous hypertension, metabolic dysfunction-associated steatotic liver disease (MASLD) with early fibrotic changes, and early-stage renal injury characterized by urine protein and renal fibrosis. Furthermore, cross-species transcriptomic analyses revealed conserved molecular signatures between the hamster model and human diabetic kidney disease. Collectively, these findings support the translational relevance of this species in modeling key aspects of human T2D and its complications.

Current T2D studies predominantly depend on rodent models, including *db/db* mice. These models are useful for modeling fundamental diabetic traits. The *db/db* golden hamster model developed in this study aims to offer distinct and clinically relevant advantages that broaden the experimental landscape of metabolic research.

T2D frequently develops as a progression of metabolic syndrome, which is frequently induced by obesity (39). In particular, *db/db* mice typically develop rapid, severe obesity very early in life (before 5 weeks), often tripling the body weight of WT littermates (16). However, obesity in patients with T2D has a variable onset, is generally less severe, and rarely reaches the extreme levels noted in young rodent models (40). In our study, *db/db* golden hamsters gained weight between 9 and 17 weeks, peaking at a rate of 24% at 14 weeks, before declining and approaching the levels noted in WT controls. In *db/db* golden hamsters, weight change was characterized by gradual and non-extreme development that was well aligned with this clinically observed human trajectory.

The lipid profile of *db/db* mice is characterized by increased total cholesterol, which is primarily induced by an increase in cholesterol. In general, the reverse lipid profile (high HDL-C and low LDL-C) in mice, combined with efficient lipid clearance, differentiates them from the dyslipidemia commonly observed in humans (37). In general, patients with T2D present with dyslipidemia, including elevated triglyceride levels and VLDL-C levels and normal or elevated LDL-C levels and total cholesterol levels (36, 41). Serum lipid profile analysis of *db/db* golden hamsters revealed dyslipidemia, with increased cholesterol and LDL-C levels, which is consistent with observations in humans.

In the context of glycemia, *db/db* mice develop early-onset hyperglycemia (at approximately 2 months of age) and exhibit a significant increase in glucose clearance. In contrast, human patients exhibit decreased glucose clearance, with hyperglycemia developing slowly and worsening over time (17, 18). Similarly, in the present study, GTT and ITT revealed that *db/db* golden hamsters exhibited impaired glucose clearance, with consistently higher glucose levels. Furthermore, the significantly elevated fasting insulin, HOMA-IR, and HbA1c levels confirm the presence of profound insulin resistance and chronic glycemic burden, which is consistent with the advanced diabetic state of this model.

Most importantly, our model successfully replicates some key complications. Patients with T2D do not succumb to hyperglycemia but to the ensuing microvascular and macrovascular complications (6, 42). Therefore, modeling these complications is paramount.

Approximately 60% of patients with T2D develop MASLD, which may progress to advanced liver disease, cirrhosis, and hepatocellular carcinoma (43, 44). We observed impaired liver function, spontaneous MASLD, and early fibrotic remodeling in *db/db* golden hamsters. While specialized dietary regimens are often required to induce similar phenotypes in mouse models (45), *db/db* hamsters offer a complementary platform to study these pathologies under spontaneous diabetic conditions. Severe lipid accumulation and FASN upregulation in *db/db* golden hamsters suggest a breakdown in lipid droplet homeostasis, a process critical to hepatic health (46). Furthermore, fibrotic stress and altered PI3K activity align with established drivers of hepatic fibrogenesis, such as the SIRT6/YAP-TAZ axis (47). Coupled with protein synthesis deficits, these findings reflect the complex proteomic and metabolic landscape of MASLD (48), positioning this model as a valuable tool for future mechanistic studies. Notably, the discrepancy between Sirius Red and Masson’s trichrome staining likely reflects the distinct spatial and morphological sensitivities of these two techniques in early-stage disease. While Sirius Red is highly sensitive to diffuse, fine interstitial collagen networks, Masson’s trichrome is particularly robust at resolving localized, pericentral, or focal extracellular matrix deposition (49–51). The presence of positive Masson signaling alongside the lack of a prominent, generalized Sirius Red change suggests that fibrotic remodeling in this spontaneous model remains restricted to an early, localized phase rather than widespread, advanced cirrhosis.

DN is a significant topic of focus in diabetes research. As a multifaceted condition, DN is primarily induced by hyperglycemia. In *db/db* mice, hyperglycemia does not uniformly develop across all individuals (11). Furthermore, this model presents limitations for investigating the contribution of hypertension to kidney injury (12). In addition, this model exhibits a less severe phenotype characterized by milder proteinuria and no significant thickening of the glomerular basement membrane (17). Renal interstitial fibrosis, a key feature and strong predictor of end-stage renal failure, is mild in *db/db* mice (52). Continuous hypertension and filtration barrier damage are the key distinguishing features of *db/db* golden hamsters, as evidenced by detectable proteinuria and a significantly elevated urinary albumin-to-creatinine ratio (ACR) at 8 weeks of age. Moreover, there is definitive evidence of renal fibrosis and glomerular basement membrane thickening, accompanied by reduced expression of podocyte markers (WT1 and nephrin) and increased deposition of collagen I and α-SMA, thereby modeling a core pathological characteristic of human DN (53, 54).

However, it is essential to acknowledge the remaining differences between *db/db* golden hamsters and human disease. Although *db/db* golden hamsters develop dyslipidemic patterns that are similar to the clinical profile, HDL-C was elevated in our model but reduced in patients with T2D (12). This paradoxical finding likely reflects the intricate, species-specific nature of lipoprotein remodeling. Unlike standard murine models that lack CETP, golden hamsters naturally express CETP (32), yielding a baseline lipid transport system that more closely mimics human physiology. Under the chronic metabolic stress of leptin receptor deficiency, the marked expansion of white adipocytes (55) and accompanying hepatic lipid overload may perturb the normal balance between HDL production and clearance, potentially contributing to the observed elevation in HDL-C (56). The exact kinetic alterations—including CETP-mediated lipid exchange and HDL turnover rates—were not quantified in the current study and await further investigation. Additionally, while emerging evidence suggests that plasma HDL-C concentration does not always correlate with functional quality (57), future studies incorporating functional assays (e.g., cholesterol efflux capacity or anti-inflammatory properties) will be performed to determine whether the quantitative HDL-C rise in our *db/db* hamsters reflects protective particles or dysfunctional subfractions.

Furthermore, although all individuals appear hyperglycemic, *db/db* golden hamsters had fasting blood glucose levels of approximately 33.3 mmol/L, a value that reflects the upper detection limit of the glucometer for some individuals, thereby exceeding the average of approximately 11.1 mmol/L in human patients (58). However, this discrepancy reflects the uncompensated, end-stage nature of the diabetic phenotype in this model, which is maintained without any glucose-lowering intervention. Importantly, the model recapitulates the temporal trajectory of T2D progression which transitions from early to advanced diabetic states within a 10-week window (8 to 18 weeks), as evidenced by the progressive deterioration of the GTT and ITT profiles (**Supplementary Figure 9**). This accelerated disease course enables the study of advanced diabetic complications within a practical experimental timeframe, offering a valuable platform for mechanistic and therapeutic investigations. Moreover, this phenotypic shift potentially reflects a transition into a decompensated phase of advanced diabetes, where severe insulin action failure may trigger progressive tissue catabolism, which closely aligns with the late-stage weight decline observed after 16 weeks of age (**Figure 1C**; **Supplementary Figure 1D**).

Additionally, several technical and experimental design limitations should be acknowledged. Blood pressure was measured under anesthesia, which may affect the absolute values. Although Avertin (tribromoethanol) has been documented to produce stable anesthesia with relatively rapid recovery in rodent models (59), it is known to influence cardiovascular parameters. Consequently, the blood pressure values reported herein may contain anesthetic artifacts; therefore, we plan to adopt conscious measurement methods in future studies. For the transcriptomic analysis, we acknowledge the inherent limitations of an exploratory RNA-seq study with sample size constraint. To ensure the robustness and reproducibility of our findings, we have implemented multiple stringent quality controls, including high RNA integrity verification, unbiased genotype-based clustering via principal component analysis, and independent qPCR validation of all representative differentially expressed genes. While these measures collectively support the validity of our current conclusions, we plan to expand the cohort size in subsequent studies to further enhance statistical robustness and capture subtler expression changes. In addition, food intake was not assessed, as our TSE system was not optimized for hamsters. Furthermore, the mechanistic studies were performed exclusively in male hamsters. Although female *db/db* hamsters exhibited similar weight trajectories, sex differences in hormonal regulation, glucose homeostasis, and insulin sensitivity have been well documented (60, 61). Therefore, future systematic characterization of female hamsters, including detailed metabolic and histological assessments, will be essential for establishing the generalizability of this model across sexes. Expanded cohorts, improved blood pressure monitoring, and comprehensive female phenotyping in future studies will further refine the phenotypic spectrum and confirm the reliability of this model under diverse experimental conditions.

In conclusion, systematic phenotyping and cross-species molecular analysis suggest that LepR deficiency in golden hamsters produces metabolic and renal changes consistent with key features of human T2D. Therefore, our developed model provides a complementary platform for mechanistic studies and the therapeutic evaluation of diabetes and its complications.

## Data Availability

The RNA-seq data have been deposited in the Gene Expression Omnibus (GEO) under accession number GSE327492.
