# Supplementary material for: A novel LepR knockout hamster model for type 2 diabetes and its complications: a complementary platform to conventional rodent models
Source: Front Endocrinol (Lausanne). 2026 Aug 14;17:1866850. doi: 10.3389/fendo.2026.1866850 (PMC13521838; doi:10.3389/fendo.2026.1866850)
Supplement: Supplementary file 1 [file SupplementaryFile1.docx]

**Supplementary Figure 1. LepR expression and metabolic characterization of *LepR* ablation (*db/db*) hamsters.**

(A) RT-qPCR analysis of *Leptin receptor (LepR)* and *Leptin* mRNA expression in various tissues of wild-type (WT) golden hamsters, mRNA levels were normalized to *β-actin* (n = 6).

(B) RT-qPCR analysis of *LepR* mRNA expression in adipose tissue, liver, and kidney in WT and *db/db* golden hamsters, mRNA levels were normalized to *β-actin* (all adjusted *p* < 0.001; n = 6).

(C) Photographs of 3-week-old WT (15 g) and *db/db* (15.6 g) golden hamsters, 8-week-old WT (142.2 g) and *db/db* (177.3 g) golden hamsters, 19-week-old WT (217.2 g) and *db/db* (209.6 g) golden hamsters, 27-week-old WT (214.3 g) and *db/db* (203 g) golden hamsters.

(D) Body weight of WT and *db/db* female golden hamsters (95% CI [-16.5, -8.03], *p* < 0.001, partial η² = 0.15; n ≥ 5).

(E-H) Serum level of leptin (E, 95% CI [5.008, 10.01], *p* = 0.003, η² = 0.9751), fasting insulin (F, 95% CI [68.56, 146.3], *p* = 0.002, η² = 0.9443) and HbA1c (G, 95% CI [28.18, 53.99], *p* = 0.003, η² = 0.9818) in 8-week-old WT and *db/db* golden hamsters (n = 3). HOMA-IR (H) was calculated as: [fasting insulin (mIU/L) × fasting glucose (mmol/L)] / 22.5; 95% CI [6.666, 53.75], *p* = 0.03, η² = 0.8680).

(I-K) Metabolic parameters in 8-week-old WT and *db/db* golden hamsters: oxygen consumption (VO₂, I), carbon dioxide production (VCO₂, J), and respiratory exchange ratio (RER, K). VO₂ and VCO₂ were measured over 24 hours, normalized to body weight, and expressed as ml/h/kg (n = 3).

Data are presented as mean ± SEM. **p* < 0.05, ***p* < 0.01, ****p* < 0.001.

**Supplementary Figure 2. Adiposity and adipocyte morphology in *db/db* hamsters.**

(A) Body fat percentage of WT and *db/db* golden hamsters (n=5, 95% CI [1.982, 4.601], *p* = 0.02, η² = 0.9241).

(B) Representative images of CD68 (red) staining in adipose tissue from WT and *db/db* golden hamsters (×4 magnification; scale bars, 250 µm). Nuclei were counterstained with DAPI (blue). Quantification of CD68-positive area (iWAT, 95% CI [0.6357, 1.018], *p* = 0.002, η² = 0.9919; eWAT, 95% CI [0.09243, 0.1716], *p* < 0.001, η² = 0.9589; 8-week-old WT and *db/db* golden hamsters, n=3).

(C) Size distribution of adipocytes of iBAT, iWAT, and eWAT (n=3).

Data are presented as mean ± SEM. **p* < 0.05, ***p* < 0.01, ****p* < 0.001.

**Supplementary Figure 3. RNA-seq profiling and validation of selected DEGs in adipose tissues.**

(A-C) PCA and correlation heatmaps of iBAT (A), iWAT (B), and eWAT (C) (8-week-old WT and *db/db* golden hamsters, n = 3).

(D-F) RNA-seq FPKM values of selected DEGs in iBAT (D), iWAT (E), and eWAT (F).

Data are presented as mean ± SEM. **p* < 0.05, ***p* < 0.01.

**Supplementary Figure 4. Macrophage infiltration in the liver of WT and *db/db* golden hamsters.**

(A) Histological staining of liver sections (Sirius red staining; ×20 magnification; scale bars, 100 µm).

(B) Representative images of CD68 (red) staining in liver from WT and *db/db* golden hamsters (×4 magnification; scale bars, 250 µm). Nuclei were counterstained with DAPI (blue). Quantification of CD68-positive area (8-week-old WT and *db/db* golden hamsters, n = 3).

Data are presented as mean ± SEM.

**Supplementary Figure 5. RNA-seq profiling and validation of selected DEGs in liver.**

(A) PCA and correlation heatmaps of liver (8-week-old WT and *db/db* golden hamsters, n = 3).

(B) RNA-seq FPKM values of selected DEGs in liver (n = 3).

Data are presented as mean ± SEM. **p* < 0.05, ***p* < 0.01, ****p* < 0.001.

**Supplementary Figure 6. Renal injury and macrophage infiltration in WT and *db/db* golden hamsters.**

(A) Representative immunofluorescence images of CD68 (red) in kidney sections (×4 magnification; scale bars, 250 µm). Nuclei were counterstained with DAPI (blue). Quantification of CD68-positive area (8-week-old WT and *db/db* golden hamsters, n = 3).

(B) Urinary microalbumin (mALB; 95% CI [50.58, 108.6], *p* = 0.002, η² = 0.9357) and urinary creatinine levels in 8-week-old WT and *db/db* golden hamsters (n = 3).

(C) Histological staining of kidney sections: renal tubules (H&E; ×20 magnification; scale bars, 100 µm).

Data are presented as mean ± SEM. ***p* < 0.01.

**Supplementary Figure 7. Immunofluorescence analysis of renal fibrosis in WT and *db/db* golden hamsters.**

Representative immunofluorescence images of Collagen I (red), Fibronectin (red), and α-SMA (red) in kidney sections (×4 magnification; scale bars, 250 µm). Nuclei were counterstained with DAPI (blue). Quantification of Collagen I-positive (95% CI [0.2869, 1.019], *p* = 0.01, η² = 0.9348), Fibronectin-positive, and α-SMA-positive areas (95% CI [0.1614, 0.7166], *p* = 0.01, η² = 0.8593; 8-week-old WT and *db/db* golden hamsters, n = 3).

Data are presented as mean ± SEM. **p* < 0.05.

**Supplementary Figure 8. RNA-seq profiling and validation of selected DEGs in kidneys.**

(A) PCA and correlation heatmaps of kidney (8-week-old WT and *db/db* golden hamsters, n=3).

(B) RNA-seq FPKM values of selected DEGs in the kidney (n=3).

Data are presented as mean ± SEM. **p* < 0.05

**Supplementary Figure 9. GTT and ITT profiles in WT and *db/db* golden hamsters at 18 weeks.**

(A) GTT (left; 95% CI [11.6, 15.9], *p* < 0.001, partial η² = 0.88) and AUC quantification (right; 95% CI [1232, 2090], *p* < 0.001, η² = 0.9666) in 18-week-old WT and *db/db* hamsters (n = 3).

(B) ITT (left; 95% CI [7.93, 15.2], *p* < 0.001, partial η² = 0.64) and AUC quantification (right; 95% CI [300.7, 2623], *p* = 0.03, η² = 0.7533) in 18-week-old WT and *db/db* hamsters (n = 3).

Data are mean ± SEM. **p* < 0.05, ***p* < 0.01, ****p* < 0.001.
