# Supplementary material for: A novel LepR knockout hamster model for type 2 diabetes and its complications: a complementary platform to conventional rodent models
Source: Front Endocrinol (Lausanne). 2026 Aug 14;17:1866850. doi: 10.3389/fendo.2026.1866850 (PMC13521838; doi:10.3389/fendo.2026.1866850)
Supplement: Supplementary file 2 [file Table1.docx]

**Supplementary Table 1.** **RT-qPCR primer list**

| Gene | Forward primer | Reverse primer |
| --- | --- | --- |
| *β-actin* | ACTGCCGCATCCTCTTCCT | TCGTTGCCAATGGTGATGAC |
| *LepR* | GTTTGGTGGATGAATCTAGCTGAGAA | ACTCTAGCACAGTCTGTGACAAGT |
| *Leptin* | TCTGCAGGACATTCTCCGAC | AGACACCTGGAAGCTAAGGC |
| *Il1β* | GGCTCCAAAAGATGAAGAGCTG | AGCTTCTCCACAGCCACAAT |
| *Il6* | TGTCTTCTTGGGACTGCTGC | CCAAACCTCCGACTTGTTGA |
| *Il10* | GGTTGCCAAACCTTATCAGAAATG | TTCACCTGTTCCACAGCCTTG |
| *Il13* | AAATGGCGGGTTCTGTGC | AATATCCTCTGGGTCTTGTAGATGG |
| *Pparγ* | CAATCAAAGTAGAACCTGCATCC | TAGTGGAAGCCTGATGCTTTATC |
| *Fabp4* | GATGTGATCACCATCCGAAC | ACCCTCACGCTTCCTCTTTA |
| *Fasn* | GCAGTCTTGAGTAGCTTTGTGCT | GGGAGCTGTCCAGATTAATACCT |
| *Atgl* | ACCATCCGTGGCTGTCTACT | AGAACGGGGACACTGTGATG |
| *Hsl* | TCTAAGTGTGTCAGTGCCT | TCCAGGAAGGAGTTGAGCC |
| *Pparα* | CCTCAGGCTACCACTACGGA | GCGTCCAAAACGAATCGCAT |
| *Dio2* | TACGGGGTAGCTTTTGAGCG | CTCCGCACTTCTTGGAGGTT |
| *Ucp1* | GAGCTGGTAACATATGACCT | TGTCCTGGCAGAGAGTTGAT |
| *Cd36* | GTATTCTCATGCCAGTTGGAGAC | TTTAACCCAGTTTTTGAAAGCAA |
| *Cox4* | AACAGGAGCACCAATGAGTG | TTGGCCTTCATGTCCAGCAT |
| *Slc25a1* | TGCATCACCTTCCCTACCGA | TCCCGAACAATCTCCCGAAC |
| *Adrb3* | TCCCCAGAAGGACTCATCGT | CGCCACCTATCGTGATCCTC |
| *Il1r2* | GTGACAAGCTCCCAGAGACAA | GGGAACTTACGGCTTTGCCA |
| *Dkk3* | ATGTTCCGAGAGGTGGAGGA | CTTGGTGCATGTGAACGGTG |
| *Lox* | GACGACCCCTACAACCCCTA | CATCCGCCCTGTATGCTGAA |
| *Serpine1* | CCTATGGACTAGGGAAAGGCTC | TATGCGGGTATGGGCTACTCA |
| *igfals* | CTTGCAGACCTTTTCGCCAC | TGTTTCACCGATGTCTCGCA |
| *Irs2* | GAGAGACAACAGACCGAGCC | AGGGGAGGCAGTTGTGTAGA |
| *Acly* | CTTAGCATTCACTAAGTTAGGCCA | ACTGTGTGAACAACCTGCT |
| *Gpam* | TCCGCGACACTGAAATGGAA | CCCTTATGGACATCCCGCTC |
| *Plin2* | GACTCTCCTGTCCAACGTCC | AAGGGACCTACCAGCCAGTT |
| *Cd68* | CTCATGTAGTTAAGCACATCGTTTC | TGTGTCAAAGTGCGTGCT |
| *Il6st* | TGTATCATAAGGGTGGGGGT | TTGCCTAAAGCCTGGCTATGT |
| *Irak4* | GGATGGAACCCCACCACTTT | TGGGTGTTATTTCTCCCCGC |
| *Gadd45a* | ATTTCACCCTCATCCAGGCG | TGTGGGTTCGTCACTAGCAC |
| *Rcan1* | AGCTTCATCGACTGCGAGAT | CTGCGGATAAGGGGTTGCTG |
| *Sod2* | CCCCAAAAGAGAGCGAGTGT | TGACTACGCCTGTTGTGGAC |
| *Atp6v1b1* | CTGGCAAACCCATCGACAAA | GCTGTCTCCATATTCACCCCC |
| *Atp10a* | CTTTATCCCCAACCACGGCT | TTGTTCTCGGTCAGAGTGCC |
| *Elovl6* | TGCACAACCTACGCAGACAG | TGATGCACAGGATTGGCTTG |
| *Klb* | AACCTGCCAACCCCTATGTG | GCTGGTTCTTGGAGGCGATA |
| *Igf1* | GCCTTAGTATGCAAGAGCCTGG | CAGTGCCCAAGAACATTTACTCC |
| *Fabp5* | CGACACAAAACCGTATGTGAAAC | CGGAAATTCATGCATATTGTTGTT |
| *Fabp1* | CTCGTCGCCACCATGAACTT | CATGCTTCCCGTTATGCTCG |
| *Elovl2* | CTACCCTGGACAGCGCATCG | GTCCAACAGGAACCACCCTC |
| *Plin1* | TCCCTTACTGGGTTCTGGGG | GGATGCAACCCATTTCGAGG |
| *plin3* | ACAACCTGAGGACTCTGTACC | GTAGGCTGCACTCACCATCTC |
| *Sdr42e1* | CCTTCCGGGACACTGACATT | TGTCTGTGCCTCTCACGTTG |
| *Akr1d1* | TGAGACGTACATGGCAGTGA | AGGATACACTTCCTTTCCAGGC |
| *Cdkn1a* | GCTGGAGGGCAACTATGTCT | CTCGTCTGTCTCCGTTTCCG |
| *Jun* | GCACTGAGCAGGAAGGATGA | GCTGGCTACGAAGAACACCC |
| *Tp53i3* | TGGAGGCCAAGCTCAGTATGT | TAATTGAATCCAGCAGCAGCC |
| *Ang* | AGACAGGGCACTTAGGACTG | TACTCAGCCACATCCTTGGAA |
| *Tnfrsf12a* | GCACTTATTTCCACATACTGTGCT | TGGAAATCTACCGGTGCAACA |
| *Rarres2* | TTGTCGCAACGGAGAAACAC | TCTAATGGTGGACCTGTGCC |
| *Vil1* | GGTAGTGCCGTCTAATCCCAG | CCTTGCTGCATCTCCTACCC |
| *Slc2a2* | AGCAGTTGATGGTCGGAAGC | GGTCCATTGATTTGGGGGTATCT |
| *Mmp7* | TTTTGCAGGGAGATTCACACA | AGGGGCTCAACCTAATGAAAC |
| *Pck1* | TCCCAGGAGCCTTGCTGTAA | AGCTTTCTATCGGGGCAAGAG |
| *Clu* | GACGCCCACTACTTCTCACC | TGCGCCTGGTGTATCATCTC |
| *Esm1* | GCCAAGTATGCGGTGGATTG | GACTGTGCGGTAGCAGGTTT |
| *Hdac9* | TGCACTGGAAGGACACATCC | AAGAGGCCCCAGCTCATTTC |
| *Myc* | GGGCTCTGCTCTCCATCCTA | GAAGGTTTCGTCGTCAGGGT |
| *Nck1* | ACTGCATGTGGTACAGGCTC | CTCAGGGTCATTTTCCGGCT |
| *Socs7* | GCAGTCTTTCCCCCTACCTC | CCCCCAATACCAACCACACT |

**Supplementary Table 2. RNA Integrity Value of all samples**

| **Sample Name** | | **RNA Integrity Value** |
| --- | --- | --- |
| WT1 | liver | 7 |
|  | kidney | 6.4 |
|  | iWAT | 6.3 |
|  | iBAT | 7.1 |
|  | eWAT | 5.4 |
| WT2 | liver | 4.5 |
|  | kidney | 5.6 |
|  | iWAT | 5.1 |
|  | iBAT | 6.8 |
|  | eWAT | 6.2 |
| WT3 | liver | 7.2 |
|  | kidney | 4.6 |
|  | iWAT | 6.4 |
|  | iBAT | 5.9 |
|  | eWAT | 6.5 |
| *LepR^-/-^*1 | liver | 6.1 |
|  | kidney | 7.6 |
|  | iWAT | 7.5 |
|  | iBAT | 6.6 |
|  | eWAT | 7 |
| *LepR^-/-^*2 | liver | 7.2 |
|  | kidney | 8.3 |
|  | iWAT | 6.4 |
|  | iBAT | 6.5 |
|  | eWAT | 6.5 |
| *LepR^-/-^*3 | liver | 6.5 |
|  | kidney | 7 |
|  | iWAT | 6.3 |
|  | iBAT | 6.4 |
|  | eWAT | 6.3 |

**Supplementary Table 3. Exact adjusted *p*-values for RT-qPCR validation of selected differentially expressed genes**

| **iBAT** | Gene | **Adjusted *p*-values** |
| --- | --- | --- |
|  | *Slc25a1* | <0.001 |
|  | *Il1r2* | 0.0002 |
|  | *Dkk3* | <0.001 |
|  | *Lox* | 0.007 |
|  | *Igfals* | <0.001 |
|  | *Irs2* | <0.001 |
| **iWAT** |  |  |
|  | *Fasn* | 0.04 |
|  | *Il6st* | 0.004 |
|  | *Lox* | 0.007 |
|  | *Serpine1* | 0.008 |
|  | *Gadd45a* | <0.001 |
| **eWAT** |  |  |
|  | *Atp10a* | <0.001 |
|  | *Cd68* | 0.02 |
|  | *Sod2* | 0.02 |
|  | *Serpine1* | 0.02 |
|  | *Igf1* | 0.04 |
| **Liver** |  |  |
|  | *Elovl6* | <0.001 |
|  | *Elovl2* | <0.001 |
|  | *Fabp4* | <0.001 |
|  | *Fabp5* | <0.001 |
|  | *Fabp1* | <0.001 |
|  | *Plin1* | <0.001 |
|  | *Sdr42e1* | 0.007 |
|  | *Akr1d1* | <0.001 |
|  | *Jun* | <0.001 |
|  | *LepR* | <0.001 |
| **Kidney** |  |  |
|  | *Tnfrsf12a* | 0.005 |
|  | *Rarres2* | <0.001 |
|  | *Vil1* | 0.009 |
|  | *Mmp7* | <0.001 |
|  | *Esm1* | <0.001 |
|  | *Hdac9* | <0.001 |
|  | *Socs7* | <0.001 |
